# Efferocytosis-associated Mrc1^+^Gas6^+^ macrophages are linked to abdominal aortic aneurysm progression through ERK-associated dysfunction

**DOI:** 10.3389/fimmu.2026.1863507

**Published:** 2026-07-07

**Authors:** Wenxuan Xiang, Xiaohong Song, Xiangyu Xian, Hao Zhou, Bingli Xu, Lei Zhou, Dan Yang, Yuehong Zheng

**Affiliations:** 1 Department of Vascular Surgery, Peking Union Medical College Hospital, Chinese Academy of Medical Sciences & Peking Union Medical College, Beijing, China; 2 Department of Vascular Surgery, The Seventh People’s Hospital of Chongqing, Chongqing, China; 3 Department of Computational Biology and Bioinformatics, Institute of Medicinal Plant Development, Chinese Academy of Medical Sciences and Peking Union Medical College, Beijing, China; 4 State Key Laboratory of Complex Severe and Rare Diseases, Peking Union Medical College Hospital, Chinese Academy of Medical Sciences and Peking Union Medical College, Beijing, China; 5 National Infrastructures for Translational Medicine, Institute of Clinical Medicine, Peking Union Medical College Hospital, Peking Union Medical College and Chinese Academy of Medical Sciences, Beijing, China

**Keywords:** abdominal aortic aneurysm, efferocytosis, ERK signaling, macrophage heterogeneity, maf, MERTK

## Abstract

**Background:**

Abdominal aortic aneurysm (AAA) is a progressive vascular disease characterized by chronic inflammation, extracellular matrix degradation, and aortic wall remodeling, yet effective pharmacological therapies remain lacking and how macrophage state heterogeneity contributes to disease progression and defective inflammation resolution remains incompletely understood.

**Methods:**

We combined single-cell RNA sequencing of elastase-induced murine AAA with pathway, cell-cell communication, trajectory, and regulon analyses, and validated key findings *in vivo* by immunostaining and flow cytometry and *in vitro* by pharmacologic ERK inhibition, gene-expression analysis, and macrophage efferocytosis assays.

**Results:**

Single-cell transcriptomic analysis identified four macrophage subsets in AAA, comprising Thbs1^+^Spp1^+^ inflammatory macrophages, Mrc1^+^Gas6^+^ efferocytosis-associated macrophages, Cdca8^+^ proliferative macrophages, and Cd36^+^Lpl^+^ lipid-handling macrophages. AAA progression was characterized by expansion of Thbs1^+^Spp1^+^ macrophages and emergence of Cdca8^+^ macrophages, together with relative loss of Mrc1^+^Gas6^+^ and Cd36^+^Lpl^+^ macrophages. Thbs1^+^Spp1^+^ macrophages showed inflammatory, chemotactic, oxidative stress, and metabolic remodeling signatures, whereas Mrc1^+^Gas6^+^ macrophages were enriched for efferocytosis- and homeostasis-associated features but exhibited increased apoptosis-related signals and reduced expression of Mertk, Gas6, and Igf1 during AAA progression. ERK signaling was overactivated in AAA and associated with loss of these effectors and impaired macrophage efferocytosis, whereas ERK inhibition restored Mertk, Gas6, and Igf1 expression and enhanced uptake of apoptotic cells in macrophage-line models. Trajectory and regulon analyses further suggested that inflammatory and efferocytosis-associated macrophages follow distinct state trajectories, with Maf emerging as a candidate regulator of the Mrc1^+^Gas6^+^ program.

**Conclusions:**

AAA is characterized by an imbalance between inflammatory and efferocytosis-associated macrophage states. ERK-associated dysfunction of Mrc1^+^Gas6^+^ macrophages may contribute to defective inflammation resolution and represents a potential therapeutic target in aneurysmal disease.

## Graphical Abstract

**Figure:**
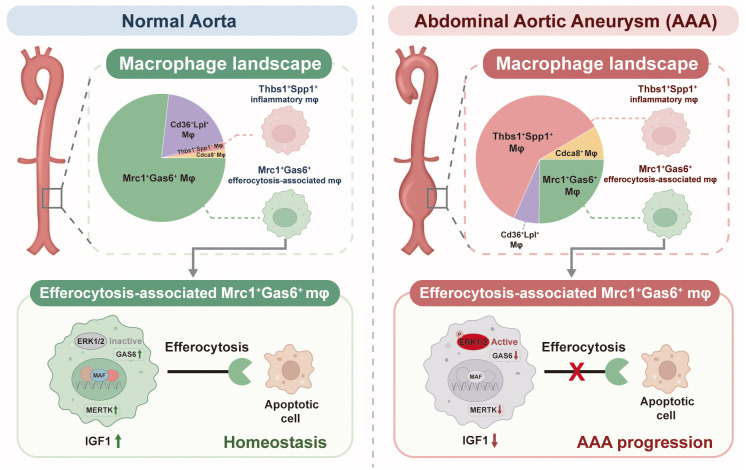

## Background

Abdominal aortic aneurysm (AAA) is a progressive and potentially life-threatening vascular disease characterized by extracellular matrix degradation, smooth muscle cell (SMC) depletion, and maladaptive aortic dilation (1, 2). Despite advances in surgical and endovascular interventions, the lack of effective pharmacological therapies to limit aneurysm expansion remains a major clinical challenge (3). It is well established that AAA is closely associated with persistent chronic inflammation within the aortic wall (4–6). As central orchestrators of the immune microenvironment, macrophages not only initiate inflammatory cascades but also participate in tissue repair and matrix remodeling (7). However, broad anti-inflammatory approaches have not yet translated into effective pharmacological therapies that reproducibly limit AAA progression, highlighting the need to better decipher the functional heterogeneity of macrophages during AAA progression (2, 3).

The classical binary “M1/M2” paradigm is increasingly recognized as insufficient to fully capture the phenotypic plasticity of macrophages *in vivo* (8–10). Recent single-cell transcriptomic studies in cardiovascular diseases, particularly atherosclerosis, have provided a more nuanced view, emphasizing functional axes such as the balance between inflammatory amplification and apoptotic cell clearance (efferocytosis) (10). Impaired efferocytosis can lead to the accumulation of apoptotic cells and secondary necrosis, thereby exacerbating inflammation and tissue injury (11). While recent single-cell analyses of AAA and related aortic disease have begun to map myeloid diversification (12–14), it remains incompletely understood how the balance between pro-inflammatory and efferocytosis-associated macrophage states is regulated in AAA, and why endogenous resolution programs are insufficient to limit aneurysm expansion.

Deciphering the regulatory networks governing these distinct macrophage states is critical for identifying potential therapeutic targets. The extracellular signal-regulated kinase (ERK) pathway, which is frequently upregulated in aneurysmal tissues, has been implicated in AAA pathogenesis (15, 16). However, emerging evidence in macrophage biology suggests that certain ERK-related pathways can regulate efferocytosis and pro-resolving responses in macrophages (17, 18). It remains to be determined whether ERK activation in AAA globally promotes inflammation or specifically influences the survival and function of distinct macrophage subpopulations. While the role of ERK signaling in macrophage state regulation in AAA remains to be clarified, the upstream transcriptional programs required to maintain efferocytosis-associated macrophages in the AAA microenvironment also remain largely undefined.

In this study, we integrated single-cell RNA sequencing with intercellular communication, trajectory, and regulon analyses, together with *in vitro* and *in vivo* validation, to characterize macrophage remodeling during elastase-induced AAA. We identified marked remodeling of macrophage states in AAA, characterized by expansion of a Thbs1^+^Spp1^+^ inflammatory subset and progressive loss of a Mrc1^+^Gas6^+^ efferocytosis-associated subset. Mechanistically, ERK activation was associated with loss of key Mrc1^+^Gas6^+^ effectors, including MerTK, Gas6, and Igf1, and with impaired apoptotic cell clearance, whereas Maf emerged as a candidate regulator associated with the Mrc1^+^Gas6^+^ program. These findings provide insight into macrophage-state dysfunction in AAA and suggest a potential link between ERK-associated signaling, impaired efferocytosis, and aneurysmal progression.

## Results

### Single-cell transcriptomic profiling reveals dynamic remodeling of macrophage heterogeneity during elastase-induced AAA formation

To define the cellular landscape of elastase-induced abdominal aortic aneurysm (AAA), we performed single-cell RNA sequencing of aortic tissues from Sham, Elastase 7d, and Elastase 14d mice. Compared with Sham controls, elastase-treated mice showed marked aortic dilation at Day 7 and Day 14 (**Supplementary Figure 1A**). The major aortic cell populations were annotated using canonical marker genes, including macrophages, smooth muscle cells (SMCs), fibroblasts (FBs), T cells, dendritic cells (DCs), endothelial cells (ECs), B cells, and a small neuronal population (**Supplementary Figure 1B**). Global UMAP visualization demonstrated marked changes in cellular composition across disease progression, with macrophages being scarce in the normal aorta but substantially accumulating in aneurysmal tissues (**Supplementary Figure 1C**).

**Figure 1:**
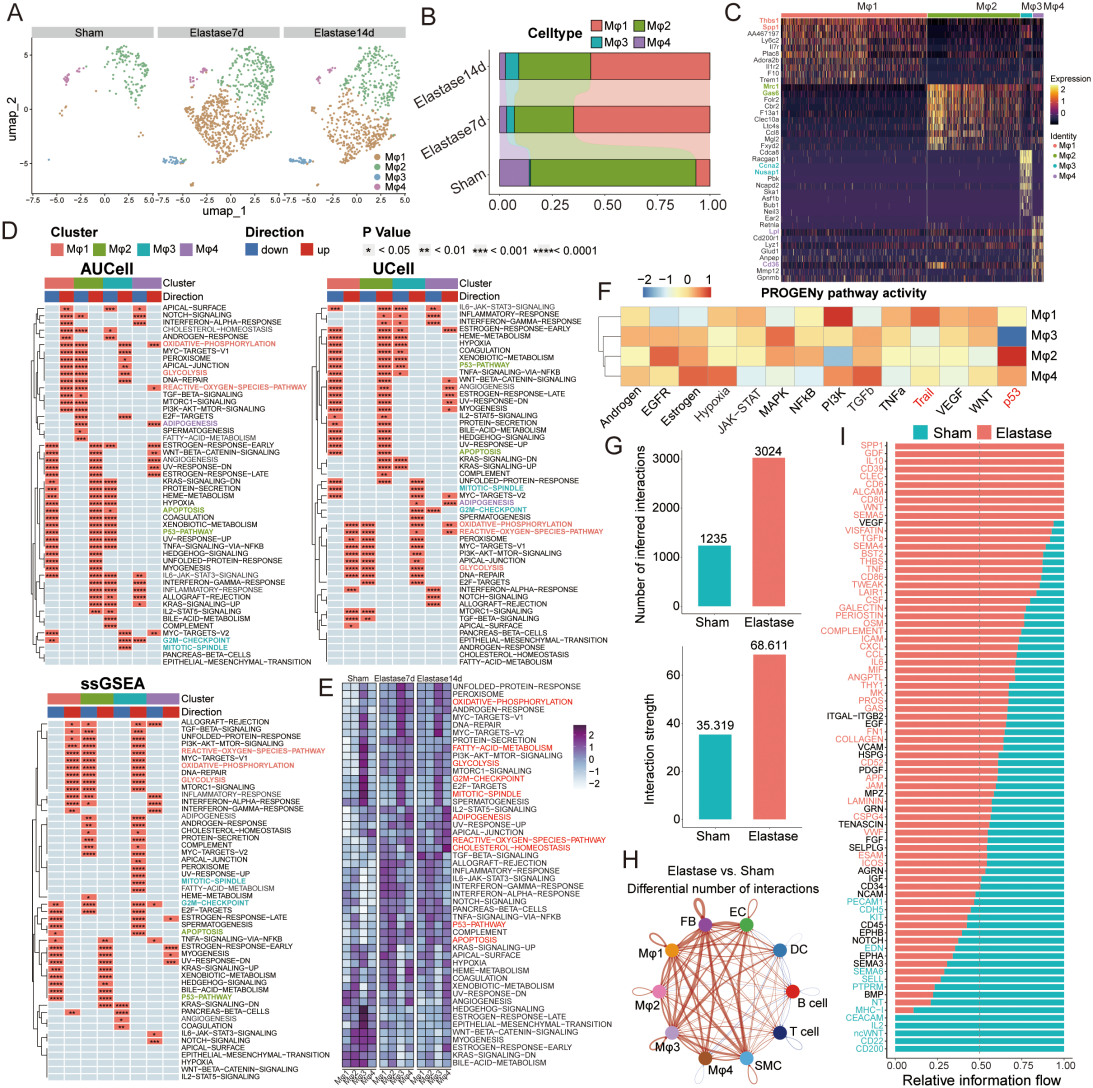
Single-cell transcriptomic analysis reveals dynamic remodeling of macrophage heterogeneity during elastase-induced AAA. Mice were grouped into Sham, Elastase 7d, and Elastase 14d. For each group, cells from infrarenal abdominal aortas were pooled from 5 mice. **(A)** UMAP visualization of re-clustered aortic macrophages stratified by group, showing four macrophage subclusters, Mφ1-Mφ4, in Sham, Elastase 7d, and Elastase 14d samples. **(B)** Stacked bar chart summarizing the relative composition of macrophage subsets in each group. **(C)** Heatmap showing the expression patterns of top 10 cluster marker genes (ordered by avg_log2FC) across Mφ1-Mφ4 subsets. **(D)** Heatmaps summarizing Hallmark pathway activity across macrophage subsets calculated using AUCell, UCell, and ssGSEA using “irGSEA” R package. Red and blue indicate relative upregulation and downregulation, respectively. **(E)** Heatmap of pathway enrichment scores across macrophage subsets in Sham, Elastase 7d, and Elastase 14d groups calculated using the “escape” R package. **(F)** Heatmap showing PROGENy-inferred pathway activity across macrophage subsets. **(G)** Bar plots showing the number of inferred intercellular interactions (upper panel) and overall interaction strength (lower panel) in Sham and Elastase samples. **(H)** Differential CellChat-inferred interaction network comparing Elastase versus Sham. Edge width indicates differential interaction number. **(I)** Relative information flow of signaling pathways inferred by CellChat in Sham and Elastase samples. Pathways with higher information flow in Elastase are shown in red, and those with relatively higher information flow in Sham are shown in cyan.

Given the prominent expansion of macrophages during AAA formation, we next reclustered the macrophage compartment and identified four transcriptionally distinct macrophage subsets, which were designated Mφ1-Mφ4 according to their relative abundance in the integrated macrophage dataset (**Figures 1A, B**). In this pooled discovery dataset, macrophages in the normal aorta were mainly composed of Mφ2 and Mφ4, whereas aneurysmal tissues showed the emergence and marked expansion of Mφ1, which became the predominant macrophage subset in both Elastase 7d and Elastase 14d lesions. A smaller Mφ3 population was also detected specifically in AAA, while the proportions of Mφ2 and Mφ4 were substantially reduced relative to Sham controls (**Figures 1A, B**). A heatmap of the top marker genes further highlighted the distinct molecular identities of these subsets: Mφ1 preferentially expressed genes such as Thbs1 and Spp1; Mφ2 was characterized by genes such as Mrc1 and Gas6; Mφ3 expressed proliferative/cell-cycle genes including Ccna2 and Nusap1; and Mφ4 preferentially expressed lipid-handling genes such as Lpl and Cd36 (**Figure 1C**).To infer the functional states of these macrophage subsets, we performed single-cell gene set activity analysis using three scoring methods implemented in irGSEA (AUCell, UCell, and ssGSEA). Across all three methods, Mφ1 consistently showed enrichment of oxidative phosphorylation, glycolysis, and reactive oxygen species (ROS)-related programs, indicating a metabolically remodeled and stress-associated state. In contrast, Mφ2 displayed comparatively lower activity of these bioenergetic pathways but higher representation of p53- and apoptosis-related programs, suggesting a relatively low-metabolic macrophage state that may be more closely associated with homeostatic functions yet vulnerable to stress-induced loss within the aneurysmal milieu. Mφ3 was strongly enriched for cell-cycle and mitotic pathways, consistent with a proliferative macrophage population, whereas Mφ4 exhibited preferential enrichment of lipid-metabolic programs, including adipogenesis (**Figure 1D**). These findings were further supported by pathway analysis using the “escape” enrichment method across disease stages, which showed that Mφ1 exhibited higher oxidative phosphorylation, glycolysis, and ROS-related signatures in aneurysmal samples; Mφ2 displayed enhanced apoptosis and p53 pathway activity; Mφ3 remained dominated by proliferative programs; and Mφ4 showed increased fatty-acid metabolism and cholesterol-homeostasis signatures (**Figure 1E**). Together, these results reveal four functionally distinct macrophage subsets and indicate substantial remodeling of macrophage composition and functional states during AAA progression.

To further infer signaling activity, we applied PROGENy analysis and found that Mφ1 was characterized by elevated TRAIL pathway activity, consistent with a pro-apoptotic signaling context, whereas Mφ2 displayed relatively high p53 pathway activity, further supporting the notion that this subset may undergo stress-associated attrition in AAA (**Figure 1F**). Notably, the combination of high metabolic flux, ROS-related programs, and death-associated signaling in Mφ1 indicates that Mφ1 might represent an inflammatory macrophage state with potential pathogenic relevance, whereas Mφ2 appears to represent a comparatively low-metabolic state that may be more closely associated with homeostatic functions but becomes constrained during aneurysm progression.

CellChat analysis further predicted a marked increase in putative intercellular communication in AAA, as reflected by both the number and overall strength of inferred interactions (**Figure 1G**). Differential interaction analysis suggested that these inferred gains were most evident between macrophages and structural vascular cells, particularly FBs, SMCs, and ECs (**Figure 1H**). At the pathway level, relative information flow analysis predicted SPP1 as the most prominently increased signaling pathway in AAA, together with higher THBS, TNF, CCL/CXCL, ICAM, complement, and TGFβ signaling, suggesting a putative increase in inflammatory and stromal-immune communication within aneurysmal tissue (**Figure 1I**). By contrast, CD200 and IGF signaling were predicted to be relatively more prominent in Sham aortas, suggesting a more homeostatic communication landscape under non-aneurysmal conditions (**Figure 1I**). Consistent with these findings, outgoing and incoming signaling analyses suggested that macrophage-centered communication was broadly intensified in AAA, with Mφ1 displaying the most pronounced increase in inferred sending and receiving activities (**Supplementary Figures 1D, E**).

Together, these data indicate that the macrophage composition of the AAA microenvironment is markedly altered, with a shift from an Mφ2/Mφ4-enriched state toward an Mφ1-centered inflammatory communication program accompanied by the emergence of proliferative Mφ3 cells.

### Thbs1^+^Spp1^+^ macrophages represent an inflammatory and metabolically remodeled subset that expands in AAA

**Figure 2:**
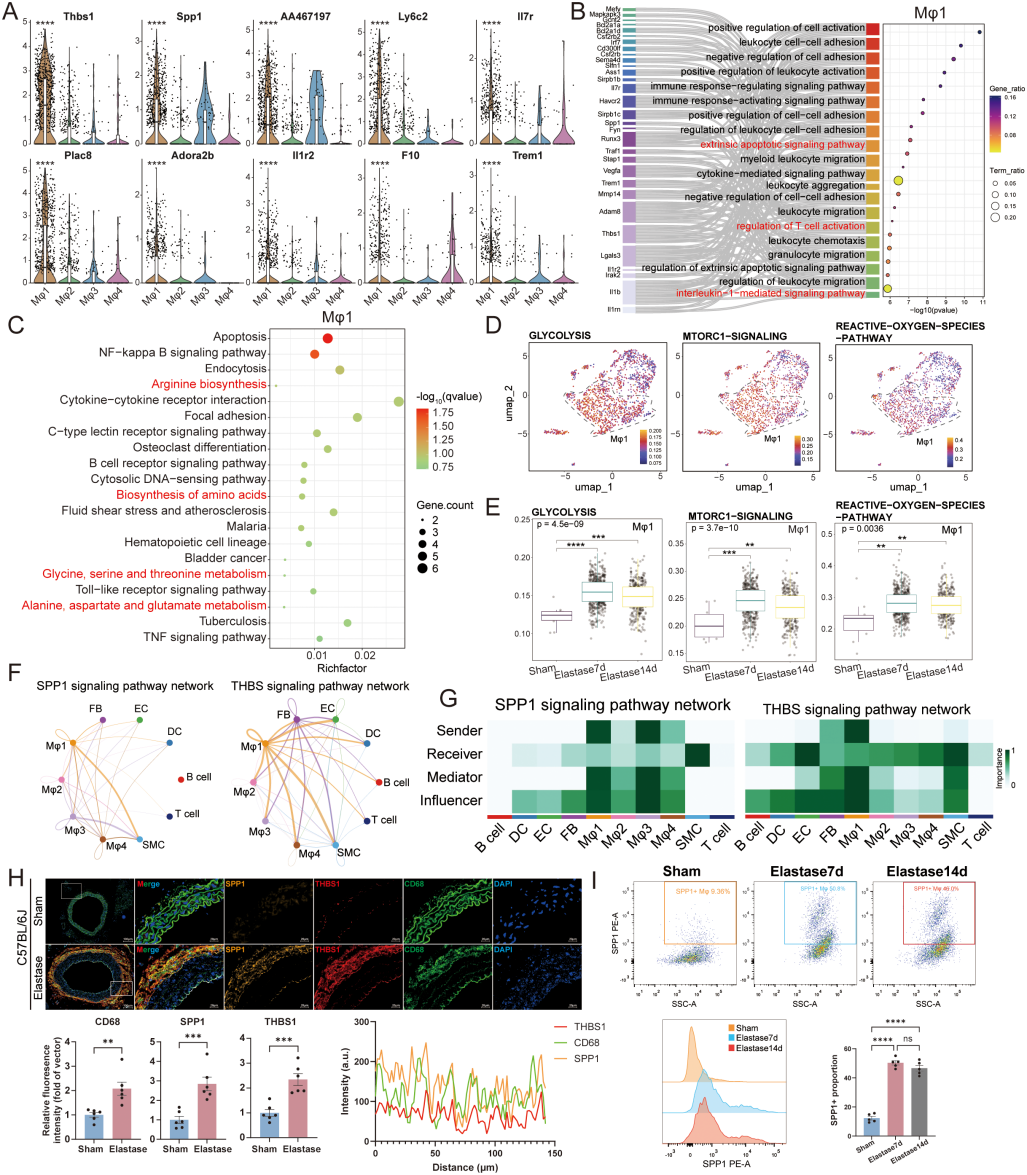
Thbs1^+^Spp1^+^ macrophages represent an inflammatory and metabolically remodeled macrophage subset in AAA. **(A)** Violin plots showing the expression levels of the top 10 Mφ1 marker genes ranked by avg_log2FC. **(B)** The top 20 enriched terms from Gene Ontology-Biological Process (GO-BP) analysis based on the top 100 Mφ1 marker genes are shown. A maximum of 8 representative genes is displayed for each enriched term. Color indicates gene ratio, and dot size indicates term ratio. **(C)** The top 20 enriched pathways from KEGG analysis based on the top 100 Mφ1 marker genes are shown. Color indicates -log_10_(q value), and dot size indicates gene count. **(D)** Feature plots showing AUCell-derived enrichment scores of glycolysis, MTORC1 signaling, and reactive oxygen species (ROS) pathway activity in macrophages. The Thbs1^+^Spp1^+^ macrophage subset is indicated in dashed lines. **(E)** Box plots showing AUCell scores of glycolysis, MTORC1 signaling, and ROS pathway activity in Mφ1 across Sham, Elastase 7d, and Elastase 14d groups. **(F)** CellChat-inferred SPP1 and THBS signaling pathway networks in Sham and Elastase samples. Edge width indicates inferred communication strength. **(G)** Signaling role analysis of the SPP1 and THBS pathways inferred by CellChat. The relative roles of each cell population as sender, receiver, mediator, and influencer are shown within the signaling network. **(H)** Representative immunofluorescence images and quantification of SPP1, THBS1 and CD68 staining in aortic tissues from Sham and Elastase-induced AAA mice (n = 6 biologically independent mice per group). Scale bar (low/high magnification): 100/20 μm. Line-scan analysis in Elastase samples shows the colocalization pattern of CD68, THBS1, and SPP1 along the selected line segment. The x-axis indicates distance along the selected line, and the y-axis indicates fluorescence intensity. **(I)** Flow cytometric analysis of SPP1-positive macrophages in aortic tissues from Sham, Elastase 7d and Elastase 14d groups. Data are presented as mean ± SEM **(E, H, I)**. Statistical significance was determined using Wilcoxon rank-sum test **(E)**, two-tailed unpaired t test **(H)**, or one-way ANOVA with Tukey’s *post hoc* test **(I)**. **P ≤ 0.01; ***P ≤ 0.001; ****P ≤ 0.0001; ns, not significant.

Based on their top marker genes, we hereafter refer to Mφ1 as Thbs1^+^Spp1^+^ macrophages. Violin plots showed that this subset was characterized by prominent expression of Thbs1 and Spp1 (**Figure 2A**). Gene Ontology analysis of the top 100 marker genes demonstrated enrichment of biological processes related to interleukin-1-mediated signaling, regulation of leukocyte migration/chemotaxis, immune response-activating signaling, and regulation of extrinsic apoptotic signaling (**Figure 2B**), indicating that this subset was associated with inflammatory activation and immune-cell recruitment. KEGG analysis further showed enrichment of Toll-like receptor signaling, NF-κB signaling, and cytokine-cytokine receptor interaction, supporting an inflammatory transcriptional profile (**Figure 2C**). Notably, this subset was also enriched for amino acid biosynthesis and metabolic pathways, suggesting additional metabolic remodeling in Thbs1^+^Spp1^+^ macrophages (**Figure 2C**).

To further assess the pathway state of this subset, we analyzed Hallmark pathway activity at single-cell resolution. Feature plots showed that glycolysis, mTORC1 signaling, and the reactive oxygen species (ROS) pathway were all preferentially enriched in Thbs1^+^Spp1^+^ macrophages (**Figure 2D**). Quantitative comparison across groups further demonstrated that these pathway scores were all elevated in aneurysmal samples relative to Sham controls (**Figure 2E**). Together, these data indicate that Thbs1^+^Spp1^+^ macrophages may represent a metabolically remodeled inflammatory subset that becomes further enhanced during AAA progression.

We next examined the communication properties of this subset. Global analysis predicted that, as a putative signaling source, Thbs1^+^Spp1^+^ macrophages preferentially interacted with macrophages, SMCs, and DCs, whereas as a putative signaling target they predominantly received inputs from SMCs and FBs (**Supplementary Figure 2A**). We then screened the CellChat pathways and found that Thbs1^+^Spp1^+^ macrophages were inferred to act as major sender populations in the SPP1 and THBS pathways (**Figures 2F, G**). In these signaling networks, SMCs and ECs were among the principal putative recipient populations (**Figures 2F, G**; **Supplementary Figure 2B**). At the ligand-receptor level, SPP1 signaling was predicted only in Elastase, and the dominant predicted ligand-receptor pair was Spp1-Cd44 (**Supplementary Figure 2C**). By contrast, the dominant THBS-associated interaction was predicted to shift from Thbs3-Cd47 in Sham to Thbs1-Cd47 in AAA (**Supplementary Figure 2C**). These findings suggest that Thbs1^+^Spp1^+^ macrophages may represent a major communication-active macrophage population in AAA.

To validate the presence of this subset *in vivo*, we performed triple immunofluorescence staining for CD68, SPP1, and THBS1 in murine aortic tissues. Immunofluorescence staining showed a marked increase in SPP1^+^THBS1^+^ macrophages in elastase-induced AAA compared with Sham aortas (**Figure 2H**). Consistently, flow cytometric analysis showed that the proportion of SPP1-positive macrophages was significantly increased in Elastase 7d and Elastase 14d aortas compared with Sham controls (**Figure 2I**). These findings support the presence and expansion of Thbs1^+^Spp1^+^ macrophages in the aneurysmal aortic wall. Overall, these data identify Thbs1^+^Spp1^+^ macrophages as an inflammatory and metabolically remodeled subset that emerges prominently in AAA and is associated with enhanced CellChat-inferred communication activity.

### Mrc1^+^Gas6^+^ macrophages represent a low-metabolic subset with apoptosis-associated features in AAA

**Figure 3:**
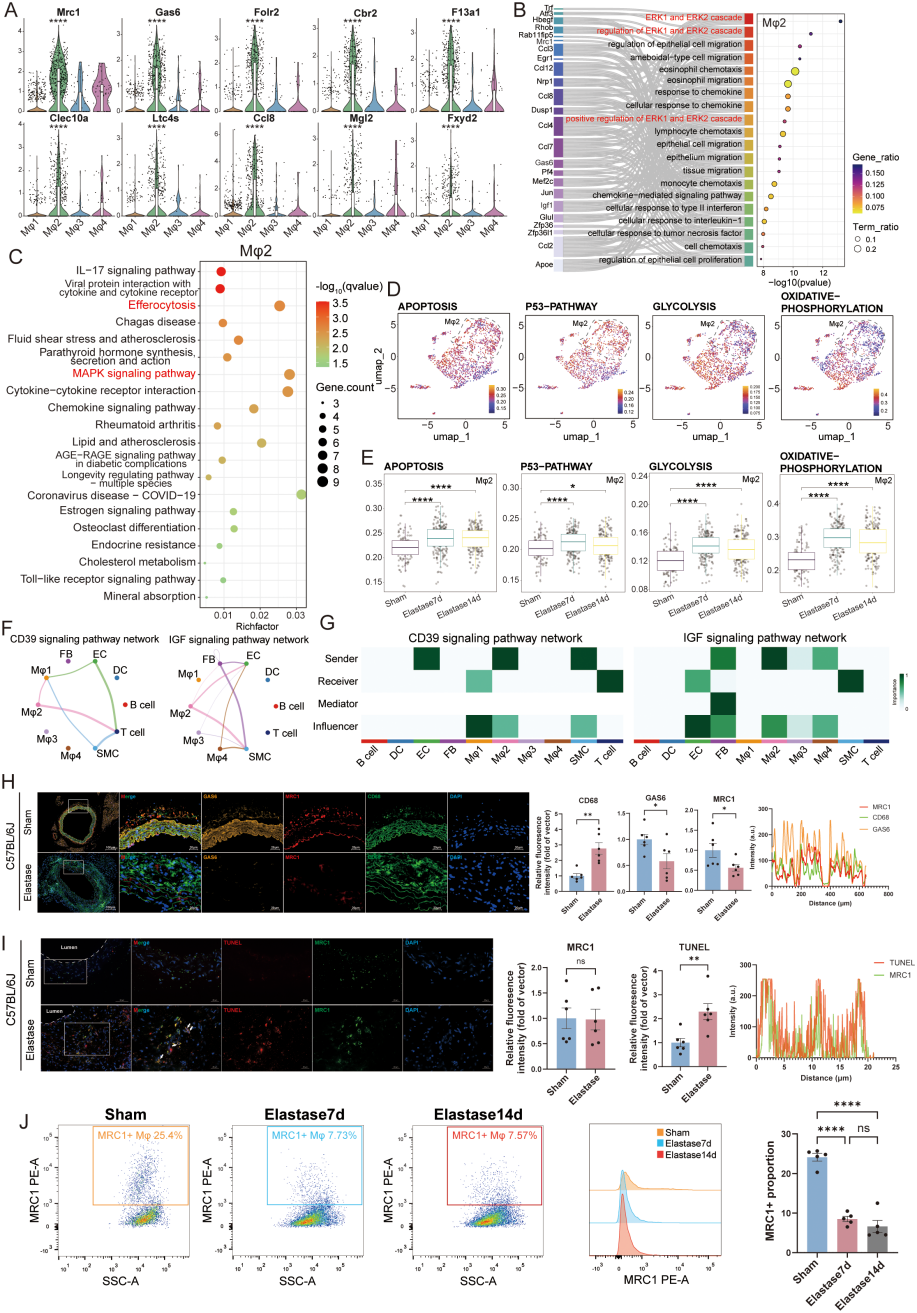
Mrc1^+^Gas6^+^ macrophages represent a homeostatic macrophage subset with candidate efferocytotic features that is progressively compromised during AAA. **(A)** Violin plots showing the expression levels of the top 10 Mφ2 marker genes ranked by avg_log2FC. **(B)** The top 20 enriched terms from GO-BP analysis based on the top 100 Mφ2 marker genes are shown. A maximum of 8 representative genes is displayed for each enriched term. Color indicates gene ratio, and dot size indicates term ratio. **(C)** The top 20 enriched pathways from KEGG analysis based on the top 100 Mφ2 marker genes are shown. Color indicates -log10(q value), and dot size indicates gene count. **(D)** Feature plots showing AUCell-derived enrichment scores of APOPTOSIS, P53_PATHWAY, GLYCOLYSIS, and OXIDATIVE_PHOSPHORYLATION in reclustered macrophages. The Mrc1^+^Gas6^+^ macrophage subset is indicated in dashed lines. **(E)** Box plots showing AUCell scores of APOPTOSIS, P53_PATHWAY, GLYCOLYSIS, and OXIDATIVE_PHOSPHORYLATION in Mφ2 across Sham, Elastase 7d, and Elastase 14d groups. **(F)** CellChat-inferred CD39 and IGF signaling pathway networks in Sham and Elastase samples. Edge width indicates inferred communication strength. **(G)** Signaling role analysis of the CD39 and IGF pathways inferred by CellChat. The relative roles of each cell population as sender, receiver, mediator, and influencer are shown within the signaling network. **(H)** Representative immunofluorescence images and quantification of MRC1, GAS6 and CD68 staining in aortic tissues from Sham and Elastase-induced AAA mice (n = 6 biologically independent mice per group). Line-scan analysis in Sham samples shows the colocalization pattern of MRC1, GAS6 and CD68 along the selected line segment. The x-axis indicates distance along the selected line, and the y-axis indicates fluorescence intensity. **(I)** Representative immunofluorescence images and quantification of TUNEL and MRC1 staining in aortic tissues from Sham and Elastase-induced AAA mice (n = 6 biologically independent mice per group). Line-scan analysis in Elastase samples shows the spatial relationship between TUNEL and MRC1 signals along the selected line segment. The x-axis indicates distance along the selected line, and the y-axis indicates fluorescence intensity. **(J)** Flow cytometric analysis of MRC1-positive macrophages in aortic tissues from Sham, Elastase 7d and Elastase 14d groups. Data are presented as mean ± SEM (E and H-J). Statistical significance was determined using Wilcoxon rank-sum test **(E)** or two-tailed unpaired t test **(H, I)**, or one-way ANOVA with Tukey’s *post hoc* test **(J)**. *P ≤ 0.05; **P ≤ 0.01; ****P ≤ 0.0001; ns, not significant.

Based on their top marker genes, we hereafter refer to Mφ2 as Mrc1^+^Gas6^+^ macrophages. Violin plots showed that this subset was characterized by prominent expression of Mrc1 and Gas6 (**Figure 3A**). Gene Ontology analysis demonstrated enrichment of biological processes related to ERK1 and ERK2 cascade, response to chemokine, and cellular response to tumor necrosis factor (**Figure 3B**), indicating an active transcriptional response to extracellular signaling cues. KEGG analysis further showed enrichment of efferocytosis, MAPK signaling, and chemokine signaling (**Figure 3C**).

To further assess the functional state of this subset, we analyzed Hallmark pathway activity. Feature plots showed that APOPTOSIS and P53_PATHWAY scores were preferentially enriched in Mrc1^+^Gas6^+^ macrophages, whereas GLYCOLYSIS and OXIDATIVE_PHOSPHORYLATION were comparatively lower in this subset (**Figure 3D**). Quantitative comparison further demonstrated that apoptotic and p53-related pathway scores were significantly increased in aneurysmal samples, whereas glycolytic and oxidative phosphorylation signatures were reduced in AAA relative to Sham controls (**Figure 3E**). Together, these data indicate that Mrc1^+^Gas6^+^ macrophages represent a relatively low-metabolic subset that becomes progressively constrained during AAA progression.

We next examined the predicted communication properties of this subset. Global analysis suggested that, as a signaling source, Mrc1^+^Gas6^+^ macrophages preferentially interacted with macrophages, whereas as a signaling target they predominantly received inputs from SMCs and FBs (**Supplementary Figure 3A**). We then identified CD39 and IGF as notable CellChat-inferred outgoing pathways from Mrc1^+^Gas6^+^ macrophages (**Figures 3F, G**; **Supplementary Figure 3B**). In aneurysmal samples, CD39 signaling from this subset was predicted to be increased and primarily directed toward Thbs1^+^Spp1^+^ macrophages and T cells, whereas IGF signaling from this subset was predicted to be overall reduced and mainly targeted ECs and SMCs (**Figures 3F, G**; **Supplementary Figure 3B**). At the ligand-receptor level, CD39 signaling was not predicted in Sham but detectable in Elastase, with Entpd1-Adora2a constituting the dominant interaction pair (**Supplementary Figure 3C**). By contrast, IGF signaling was present in both Sham and Elastase, mainly represented by the Igf1-Igf1r pair (**Supplementary Figure 3C**).

To validate the Mrc1^+^Gas6^+^ macrophage subset at the protein level, we performed triple immunofluorescence staining for CD68, MRC1, and GAS6. MRC1^+^GAS6^+^ macrophages were detectable in Sham aortas but were reduced in elastase-induced AAA tissues (**Figure 3H**). Because apoptosis-related programs were enriched in this subset, we further performed TUNEL staining and found that TUNEL-positive MRC1-positive cells were markedly increased in aneurysmal tissues compared with Sham controls (**Figure 3I**). Consistent with this observation, flow cytometric analysis showed that the proportion of MRC1-positive macrophages was significantly reduced in Elastase 7d and Elastase 14d aortas compared with Sham controls (**Figure 3J**). Overall, these data identify Mrc1^+^Gas6^+^ macrophages as a low-metabolic subset that is present in the normal aorta but becomes progressively reduced during AAA, accompanied by increased apoptosis-associated features and altered predicted intercellular signaling.

### Cdca8^+^ macrophages represent a proliferative subset enriched in AAA

**Figure 4:**
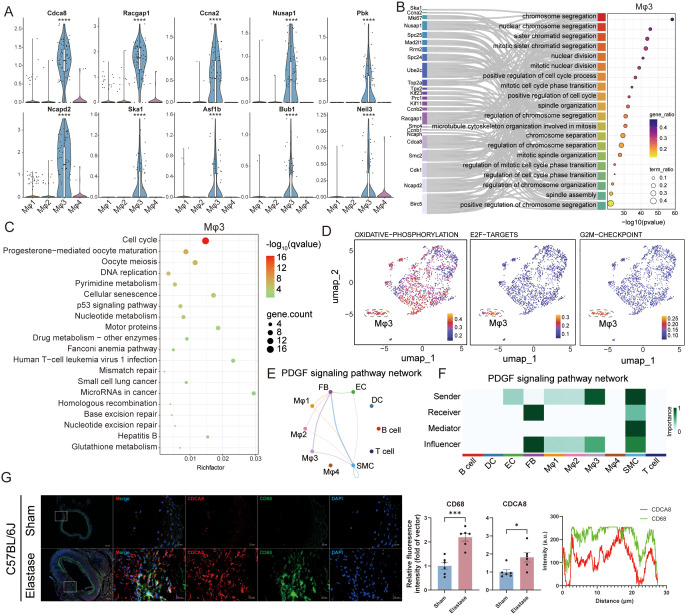
Cdca8^+^ macrophages represent a proliferative macrophage subset enriched in AAA. **(A)** Violin plots showing the expression levels of the top 10 Cdca8^+^ macrophage marker genes ranked by avg_log2FC. **(B)** The top 20 enriched terms from GO-BP analysis based on the top 100 Cdca8^+^ macrophage marker genes are shown. A maximum of 8 representative genes is displayed for each enriched term. Color indicates gene ratio, and dot size indicates term ratio. **(C)** The top 20 enriched pathways from KEGG analysis based on the top 100 Cdca8^+^ macrophage marker genes are shown. Color indicates -log10(q value), and dot size indicates gene count. **(D)** Feature plots showing AUCell enrichment scores of OXIDATIVE_PHOSPHORYLATION, E2F_TARGETS, and G2M_CHECKPOINT in re-clustered macrophages. The Cdca8^+^ macrophage subset is indicated in dashed lines. **(E)** CellChat-inferred PDGF signaling pathway networks in Sham and Elastase samples. Edge width indicates inferred communication strength. **(F)** Signaling role analysis of the PDGF pathway inferred by CellChat. The relative roles of each cell population as sender, receiver, mediator, and influencer are shown within the signaling network. **(G)** Representative immunofluorescence images and quantification of CDCA8 and CD68 staining in aortic tissues from Sham and Elastase-induced AAA mice (n = 6 biologically independent mice per group). Line-scan analysis in Elastase samples shows the spatial relationship between CDCA8 and CD68 signals along the selected line segment. The x-axis indicates distance along the selected line, and the y-axis indicates fluorescence intensity. Data are presented as mean ± SEM **(G)**. Statistical significance was determined using two-tailed unpaired t test **(G)**. *P ≤ 0.05; ***P ≤ 0.001; ****P ≤ 0.0001.

Based on their top marker genes, we hereafter refer to Mφ3 as Cdca8^+^ macrophages. Violin plots showed that this subset was characterized by prominent expression of Cdca8 (**Figure 4A**). Gene Ontology analysis demonstrated strong enrichment of biological processes related to chromosome segregation, sister chromatid segregation, nuclear division, mitotic spindle assembly, and regulation of mitotic cell cycle phase transition (**Figure 4B**). Consistently, KEGG analysis showed enrichment of cell cycle, DNA replication, and other cell division-associated pathways (**Figure 4C**). Together, these findings identify Cdca8^+^ macrophages as a subset with a dominant proliferative transcriptional program.

Hallmark pathway activity analysis showed that OXIDATIVE_PHOSPHORYLATION, E2F_TARGETS, and G2M_CHECKPOINT scores were preferentially enriched in Cdca8^+^ macrophages (**Figure 4D**). These data indicate that this subset is characterized not only by cell-cycle activation but also by enhanced energy metabolism. Intercellular communication analysis predicted that, as a putative signaling source, Cdca8^+^ macrophages primarily communicated with macrophage populations, whereas they mainly received signals from FBs and SMCs (**Supplementary Figure 4A**). We identified PDGF as a notable CellChat-inferred pathway in which Cdca8^+^ macrophages were predicted to serve as one of the major senders, with FBs and SMCs acting as recipient populations (**Figures 4E, F**). At the ligand-receptor level, major predicted interaction pairs included Pdgfa-Pdgfra and Pdgfa-Pdgfrb (**Supplementary Figure 4C**).

To validate the presence of this subset *in vivo*, we examined CDCA8 expression in murine aortic tissues. Immunofluorescence staining showed that CDCA8-positive CD68-positive cells were increased in elastase-induced AAA compared with Sham aortas (**Figure 4G**). Overall, these data identify Cdca8^+^ macrophages as a proliferative subset enriched in AAA, characterized by strong cell-cycle activity, increased oxidative phosphorylation, and PDGF-associated intercellular communication.

### Cd36^+^Lpl^+^ macrophages represent a lipid-handling subset associated with CD200 signaling

**Figure 5:**
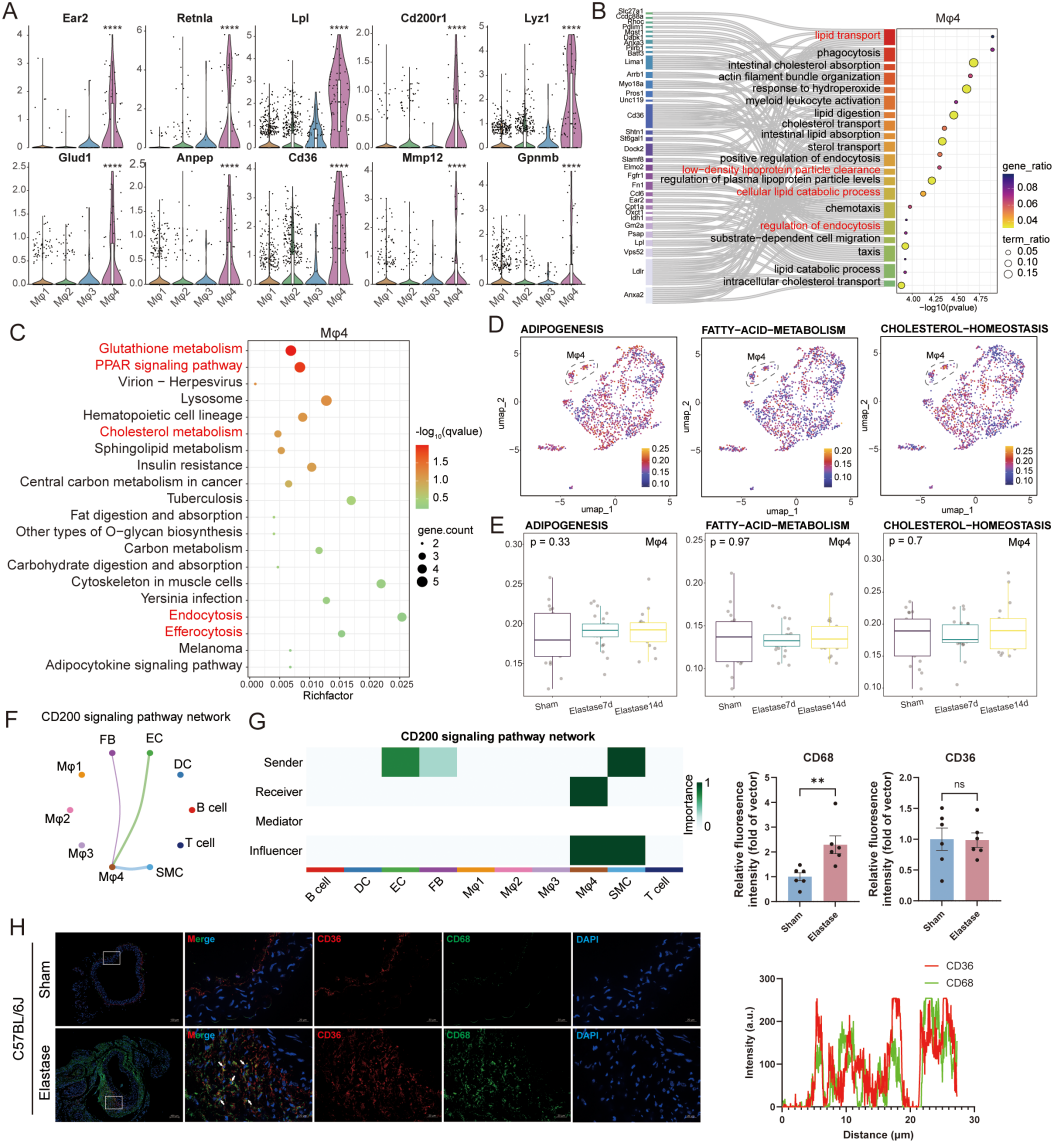
Cd36^+^Lpl^+^ macrophages represent a lipid-handling subset associated with CD200 signaling. **(A)** Violin plots showing the expression levels of the top 10 Cd36^+^Lpl^+^ macrophage marker genes ranked by avg_log2FC. **(B)** The top 20 enriched terms from GO-BP analysis based on the top 100 Cd36^+^Lpl^+^ macrophage marker genes are shown. A maximum of 8 representative genes is displayed for each enriched term. Color indicates gene ratio, and dot size indicates term ratio. **(C)** The top 20 enriched pathways from KEGG analysis based on the top 100 Cd36^+^Lpl^+^ macrophage marker genes are shown. Color indicates -log10(q value), and dot size indicates gene count. **(D)** Feature plots showing AUCell enrichment scores of ADIPOGENESIS, FATTY_ACID_METABOLISM, and CHOLESTEROL_HOMEOSTASIS in re-clustered macrophages. The Cd36^+^Lpl^+^ macrophage subset is indicated in dashed lines. **(E)** Box plots showing AUCell scores of ADIPOGENESIS, FATTY_ACID_METABOLISM, and CHOLESTEROL_HOMEOSTASIS in Cd36^+^Lpl^+^ macrophages across Sham, Elastase 7d, and Elastase 14d groups. **(F)** CellChat-inferred CD200 signaling pathway networks in Sham and Elastase samples. Edge width indicates inferred communication strength. **(G)** Signaling role analysis of the CD200 pathway inferred by CellChat. The relative roles of each cell population as sender, receiver, mediator, and influencer are shown within the signaling network. **(H)** Representative immunofluorescence images and quantification of CD36 and CD68 staining in aortic tissues from Sham and Elastase-induced AAA mice (n = 6 biologically independent mice per group). Line-scan analysis in Sham samples shows the spatial relationship between CD36 and CD68 signals along the selected line segment. The x-axis indicates distance along the selected line, and the y-axis indicates fluorescence intensity. Data are presented as mean ± SEM **(E, H)**. Statistical significance was determined using Wilcoxon rank-sum test **(E)** or two-tailed unpaired t test **(H)**. **P ≤ 0.01; ****P ≤ 0.0001; ns, not significant.

Based on their top marker genes, we hereafter refer to Mφ4 as Cd36^+^Lpl^+^ macrophages. Violin plots showed prominent expression of Cd36 and Lpl (**Figure 5A**). Gene Ontology analysis demonstrated enrichment of biological processes related to lipid transport, lipid catabolic process, regulation of endocytosis, and low-density lipoprotein particle clearance (**Figure 5B**). KEGG analysis further showed enrichment of cholesterol metabolism, PPAR signaling, glutathione metabolism, and endocytosis (**Figure 5C**), supporting a lipid-associated macrophage state.

Feature plots showed that ADIPOGENESIS, FATTY_ACID_METABOLISM, and CHOLESTEROL_HOMEOSTASIS were preferentially enriched in Cd36^+^Lpl^+^ macrophages (**Figure 5D**). Quantitative comparison across groups showed no significant differences in these pathway scores among Sham, Elastase 7d, and Elastase 14d samples (**Figure 5E**), suggesting that the lipid-metabolic features of this subset were stably maintained across disease stages. Communication analysis suggested CD200 as a notable pathway associated with this subset, wherein Cd36^+^Lpl^+^ macrophages were predicted to act as a major receiver (**Figures 5F, G**). The dominant predicted interaction within this pathway was Cd200-Cd200r1 (**Supplementary Figure 5C**). Immunofluorescence staining confirmed that CD36-positive CD68-positive cells were detectable in both Sham and elastase-induced AAA aortas (**Figure 5H**). Overall, these data identify Cd36^+^Lpl^+^ macrophages as a macrophage subset with lipid-metabolic features and CellChat-inferred CD200-associated intercellular communication patterns.

### Inflammatory and efferocytosis-associated macrophage programs diverge in AAA, with ERK activation linked to loss of Mrc1^+^Gas6^+^ macrophage effectors

**Figure 6:**
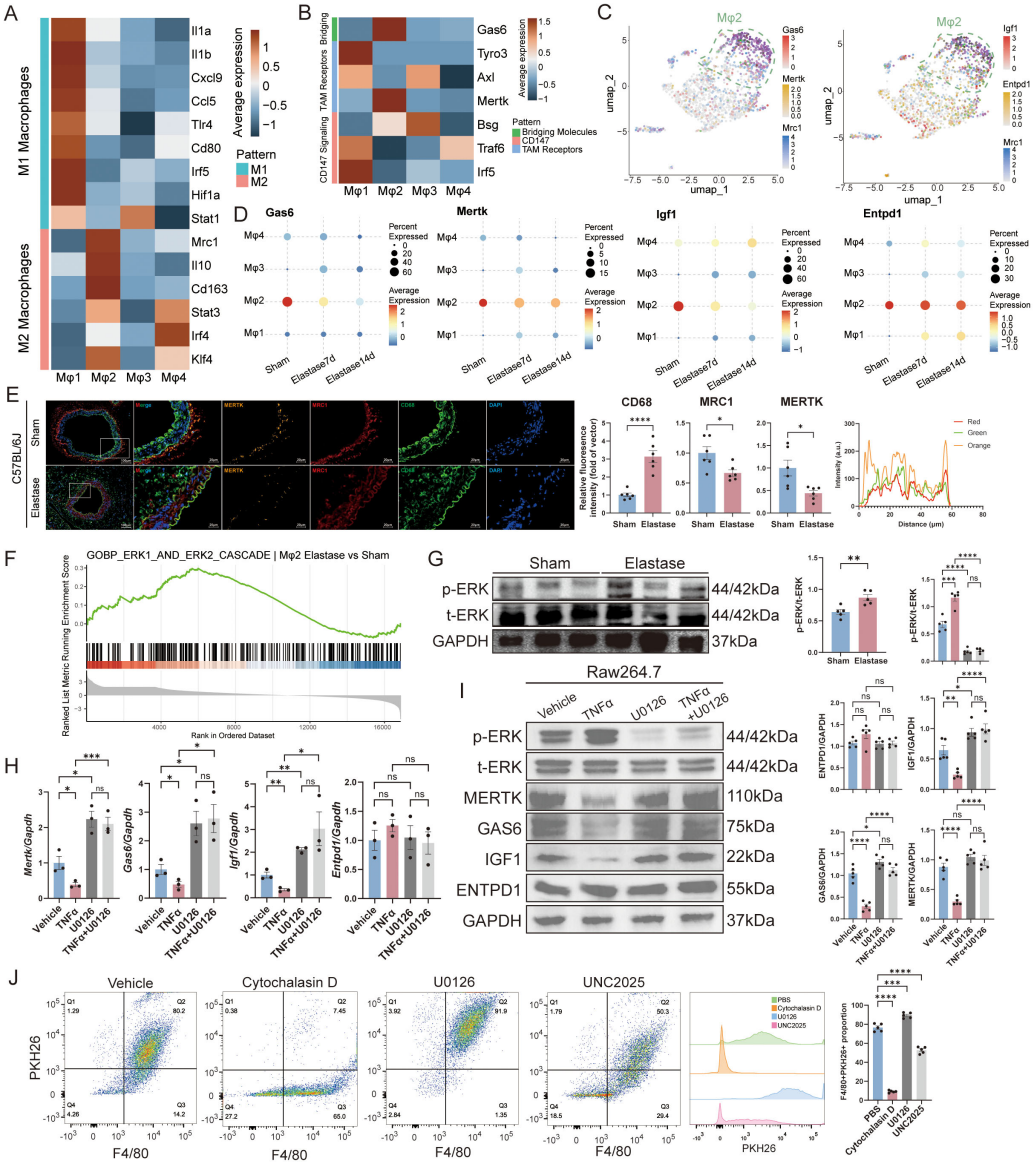
ERK activation is associated with loss of Mrc1^+^Gas6^+^ macrophage effectors and reduced macrophage efferocytosis-associated uptake. **(A)** Heatmap showing the average expression of representative M1- and M2-associated macrophage markers across Mφ1-Mφ4 subsets. The color scale indicates average expression. **(B)** Heatmap showing the average expression of efferocytosis-related molecules across macrophage subsets, including the bridging molecule Gas6, the TAM receptors Tyro3, Axl, and Mertk, and the CD147-associated molecules Bsg, Traf6, and Irf5. The color scale indicates average expression. **(C)** Feature plots showing the expression patterns of Gas6, Mertk, and Mrc1 (left), and Igf1, Entpd1, and Mrc1 (right) in re-clustered macrophages. The Mrc1^+^Gas6^+^ macrophage subset is indicated in dashed line. **(D)** Dot plots showing the expression levels and proportions of Gas6, Mertk, Igf1, and Entpd1 across macrophage subsets in Sham, Elastase 7d, and Elastase 14d groups. Dot size indicates the fraction of cells expressing the indicated gene, and color indicates the average expression level. **(E)** Representative immunofluorescence images and quantification of MRC1, MERTK and CD68 staining in aortic tissues from Sham, and Elastase AAA mice (n = 6 biologically independent mice per group). Scale bar (low/high magnification): 100/20 μm. Line-scan analysis in Sham samples shows the colocalization pattern of CD68, MRC1, and MERTK along the selected line segment. The x-axis indicates distance along the selected line, and the y-axis indicates fluorescence intensity. **(F)** Gene set enrichment analysis (GSEA) showing enrichment of the ERK1_AND_ERK2_CASCADE signature in Mrc1^+^Gas6^+^ macrophages from Elastase relative to Sham samples. **(G)** Representative western blot images and quantification of p-ERK and t-ERK protein levels in aortic tissues from Sham and Elastase-induced AAA mice. (n = 5 biologically independent mice per group). **(H)** qPCR analysis of Mertk, Igf1, Gas6, and Entpd1 mRNA levels in RAW264.7 macrophages treated with vehicle, TNF-α, U0126, or TNF-α plus U0126 (n = 3 biologically independent experiments). **(I)** Representative western blot images and quantification of p-ERK, t-ERK, MERTK, GAS6, IGF1, and ENTPD1 protein levels in RAW264.7 macrophages treated with vehicle, TNF-α, U0126, or TNF-α plus U0126. (n = 5 biologically independent experiments; quantitative comparisons between samples were run on the same gel). **(J)** Flow cytometric analysis of macrophage efferocytosis-associated uptake. RAW264.7 macrophages were co-cultured with apoptotic PKH26-labeled SMCs in the presence of vehicle, cytochalasin D, U0126, or the MERTK inhibitor UNC2025. Representative flow cytometry plots, PKH26 histograms, and quantification of the proportion of F4/80-positive PKH26-positive macrophages are shown (n = 5 biologically independent experiments). Data are presented as mean ± SEM **(E, G–J)**. Statistical significance was determined using two-tailed unpaired t test **(E, G)** or one-way ANOVA with Tukey’s *post hoc* test **(H–J)**. *P ≤ 0.05; **P ≤ 0.01; ***P ≤ 0.001; ****P ≤ 0.0001; ns, not significant.

Because our single-cell analysis identified Thbs1^+^Spp1^+^ macrophages as an inflammatory subset and Mrc1^+^Gas6^+^ macrophages as a subset enriched for efferocytosis-related features, we next examined this functional dichotomy in greater detail. We first assessed classical M1/M2-associated marker expression across the four macrophage subsets. M1-associated markers were predominantly enriched in Thbs1^+^Spp1^+^ macrophages, whereas M2-associated markers were mainly enriched in Mrc1^+^Gas6^+^ macrophages (**Figure 6A**). We then examined key molecules implicated in efferocytosis, including the bridging molecule Gas6, the TAM receptors (Mertk, Axl, and Tyro3), and the CD147/Bsg signaling complex (10). Among these molecules, Gas6 and Mertk were most prominently enriched in Mrc1^+^Gas6^+^ macrophages, whereas Axl and Tyro3 were not preferentially expressed in this subset (**Figure 6B**). These data identify Mrc1^+^Gas6^+^ macrophages as the subset most closely associated with a candidate Gas6-MerTK efferocytosis-related program in AAA.

Feature plots further showed that Gas6, Mertk, Igf1, and Entpd1 were co-expressed with Mrc1 in the Mrc1^+^Gas6^+^ cluster (**Figure 6C**). Dot plots confirmed that these genes were mainly expressed in this subset, and further showed that Gas6, Mertk, and Igf1 were markedly reduced in aneurysmal samples relative to Sham controls, whereas Entpd1 remained comparatively stable (**Figure 6D**). Together, these findings indicate that multiple efferocytosis- and homeostasis-associated molecules within Mrc1^+^Gas6^+^ macrophages are selectively diminished during AAA progression.

To further evaluate whether MERTK protein is associated with MRC1^+^ macrophages *in vivo*, we performed immunofluorescence staining for CD68, MRC1, and MERTK. MERTK signal was detectable in MRC1^+^CD68^+^ macrophages in Sham aortas but was reduced in elastase-induced AAA tissues (**Figure 6E**). These data provide protein-level support for the presence of a MRC1^+^MERTK^+^ macrophage population and are consistent with the transcriptomic enrichment of the Gas6–MerTK efferocytosis-associated program in Mrc1^+^Gas6^+^ macrophages.

Because our earlier enrichment analysis showed that Mrc1^+^Gas6^+^ macrophages were prominently associated with the ERK1/2 pathway, we next examined whether ERK signaling might be linked to this phenotypic shift. GSEA demonstrated that the ERK1 and ERK2 cascade was positively enriched in Mrc1^+^Gas6^+^ macrophages from Elastase relative to Sham samples (**Figure 6F**). Consistently, western blot analysis of aortic tissues showed that ERK phosphorylation (p-ERK) was significantly increased in elastase-induced AAA compared with Sham controls (**Figure 6G**).

To explore whether ERK signaling regulates the expression of Mrc1^+^Gas6^+^ macrophage-associated molecules, we treated RAW264.7 macrophages with vehicle, TNF-α, U0126, or TNF-α plus U0126. qPCR analysis showed that TNF-α reduced *Mertk*, *Igf1*, and *Gas6* mRNA levels, whereas U0126 restored or increased their expression under both basal and TNF-α-stimulated conditions; Entpd1 did not show a consistent ERK-dependent change (**Figure 6H**). Western blot analysis further confirmed that U0126 inhibited ERK phosphorylation and concomitantly increased MERTK, GAS6, and IGF1 protein expression, while ENTPD1 remained unchanged (**Figure 6I**). These findings suggest that Mertk, Gas6, and Igf1, but not Entpd1, are subjected to ERK-associated regulation in macrophage-line models. To further evaluate whether this effect was reproducible in an independent macrophage model, we performed parallel experiments in PMA-differentiated THP-1 macrophages. Consistent with the RAW264.7 results, U0126 reduced ERK phosphorylation and increased MERTK, GAS6, and IGF1 expression at both mRNA and protein levels under basal and TNF-α-stimulated conditions, while ENTPD1 remained largely unchanged (**Supplementary Figures 6A, B**). These results suggest that ERK inhibition can preserve or restore a MERTK/GAS6/IGF1-associated efferocytosis-related program in macrophage-line models.

We next examined whether ERK signaling influences macrophage efferocytosis-associated uptake. RAW264.7 macrophages were co-cultured with apoptotic PKH26-labeled SMCs, and uptake was quantified by flow cytometric detection of F4/80-positive PKH26-positive macrophages. Before the efferocytosis assay, apoptosis induction in PKH26-labeled SMCs was validated by Annexin V/PI staining (**Supplementary Figure 6C**). PKH26-labeled apoptotic SMCs were then co-cultured with macrophages at an apoptotic SMC-to-macrophage ratio of 3:1. ERK inhibition with U0126 significantly increased the proportion of F4/80^+^PKH26^+^ macrophages, whereas pharmacologic inhibition of MERTK with UNC2025 significantly reduced this proportion (**Figure 6J**). Cytochalasin D markedly reduced F4/80^+^PKH26^+^ events, supporting that the measured signal largely reflected actin-dependent engulfment-associated uptake rather than passive association alone (**Figure 6J**). Overall, these data suggest that AAA is accompanied by an imbalance between inflammatory and efferocytosis-associated macrophage programs, and identify ERK activation as a pathway linked to the loss of Mrc1^+^Gas6^+^ macrophage effectors and impaired macrophage efferocytosis-associated uptake.

### Inflammatory and efferocytosis-associated macrophages show distinct pseudotime-associated transcriptional patterns, with Maf associated with the Mrc1^+^Gas6^+^ program

**Figure 7:**
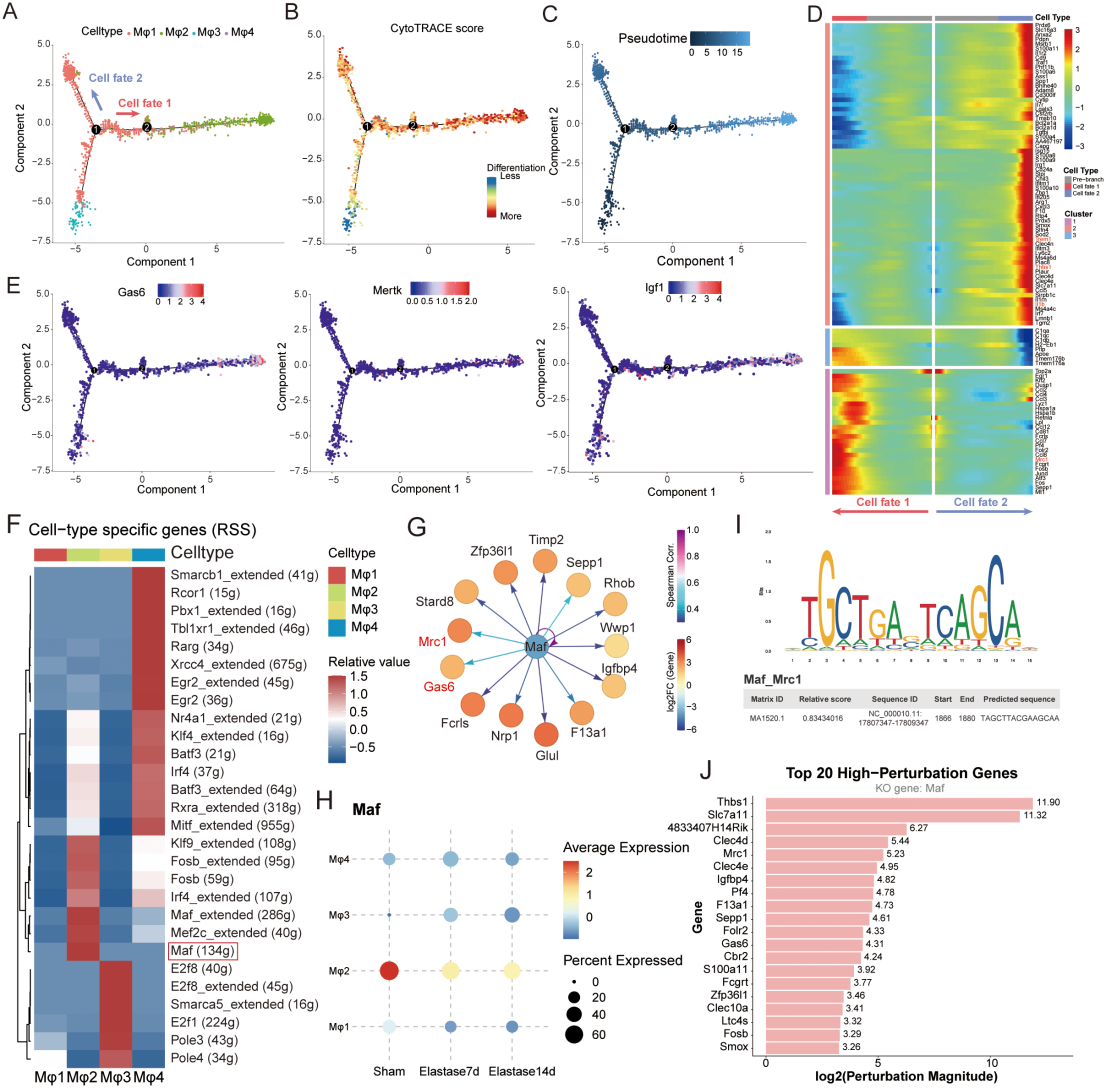
Inflammatory and efferocytosis-associated macrophages show distinct pseudotime-associated transcriptional patterns, with Maf associated with the Mrc1^+^Gas6^+^ program. **(A)** Trajectory plot of macrophages showing the inferred trajectories of the four macrophage subsets. Cells are colored by macrophage subtype. **(B)** CytoTRACE2 scores of macrophage subsets. Higher scores indicate greater inferred developmental potential. **(C)** Trajectory plot of macrophages colored by pseudotime. **(D)** Branched heatmap showing dynamic gene expression changes associated with the branch point 1 of the macrophage trajectory. The branch ending in Mrc1^+^Gas6^+^ macrophages is labeled as Cell fate 1, and the branch ending in Thbs1^+^Spp1^+^ macrophages is labeled as Cell fate 2. **(E)** Feature plots showing the expression patterns of representative inflammatory and efferocytosis-associated genes along the macrophage trajectory, including Gas6, Mertk, and Igf1. **(F)** Heatmap of regulon specificity scores (RSS) across macrophage subsets generated by SCENIC analysis. Color scale represents the relative regulon activity across macrophage subsets. **(G)** Network visualization of the Maf regulon predicted by SCENIC analysis. Nodes represent Maf and its predicted target genes, colored by log2 fold change. Edge color indicates the strength of Spearman correlation. **(H)** Dot plot showing the expression levels and proportions of Maf across macrophage subsets in Sham, Elastase 7d, and Elastase 14d groups. Dot size indicates the fraction of cells expressing Maf, and color indicates the average expression level. **(I)** In silico prediction of Maf-binding sites within the upstream 2kb promoter regions of Mrc1 using JASPAR. The highest-scoring predicted Maf-binding motif is shown. **(J)** Top 20 high-perturbation genes identified by in silico Maf knockout analysis in macrophages using the sctenifoldknk package. The x-axis indicates log2 perturbation magnitude.

To explore the transcriptional-state organization of inflammatory (Thbs1^+^Spp1^+^) and efferocytosis-associated (Mrc1^+^Gas6^+^) subsets within the macrophage compartment, we performed pseudotime trajectory analysis. The trajectory map showed that Mrc1^+^Gas6^+^ macrophages were mainly positioned at the terminus of one branch, whereas the opposite branch terminated predominantly in Thbs1^+^Spp1^+^ macrophages (**Figure 7A**). To identify a plausible starting population for trajectory inference, we calculated CytoTRACE2 scores across subsets and found that Cdca8^+^ macrophages had the highest inferred developmental potential (**Figure 7B**). Given this result, together with their proliferative features, Cdca8^+^ macrophages were selected as a plausible computational root for pseudotime ordering. The resulting pseudotime map showed progressive separation of macrophages along two major trajectories (**Figure 7C**).

To define transcriptional changes along these trajectories, we examined the top pseudotime-associated genes. A heatmap showed that Mrc1, Igf1, and Gas6 clustered together and were predominantly expressed toward the terminal portion of pseudotime (**Supplementary Figure 7A**). Branch-dependent analysis at the major bifurcation further demonstrated that the branch ending in Mrc1^+^Gas6^+^ macrophages retained high Mrc1 expression, whereas the branch ending in Thbs1^+^Spp1^+^ macrophages showed progressive upregulation of Trem1, Thbs1, and Il1b along pseudotime (**Figure 7D**). Consistently, projection of representative genes onto the trajectory map showed that Il1b and Thbs1 were concentrated at the terminal region of the inflammatory branch, whereas Gas6, Mertk, Igf1, and Mrc1 were enriched at the terminal region of the alternative branch (**Figure 7E**; **Supplementary Figure 7B**). Together, these data suggest that macrophages in AAA can be computationally organized into two major pseudotime-associated transcriptional patterns, one terminating in an inflammatory program and the other in a Mrc1^+^Gas6^+^ homeostatic program.

To explore transcriptional regulators associated with the Mrc1^+^Gas6^+^ trajectory, we performed SCENIC analysis. Regulon specificity analysis identified Maf as one of the most prominent transcription factors enriched in Mrc1^+^Gas6^+^ macrophages (**Figure 7F**). SCENIC also predicted Gas6 and Mrc1 as candidate downstream targets within the Maf-associated regulon (**Figure 7G**). Consistent with this, Maf displayed expression dynamics similar to those of the Mrc1^+^Gas6^+^ program in pseudotime analysis (**Supplementary Figure 7A**) and was enriched at the terminal region of the same branch (**Supplementary Figure 7B**). Dot plot analysis further showed that Maf was preferentially expressed in Mrc1^+^Gas6^+^ macrophages and was reduced in aneurysmal samples compared with Sham controls (**Figure 7H**).

To further evaluate whether Maf may regulate Mrc1^+^Gas6^+^ macrophage-associated genes, we used JASPAR to predict Maf-binding motifs within the upstream promoter regions of Mrc1, Gas6, Mertk, and Igf1. Predicted Maf-binding sites were identified in all four genes, with the highest-scoring candidate sites shown in **Figure 7I**; **Supplementary Figure 7C**. We then performed in silico Maf knockout analysis in macrophages and found that Mrc1 and Gas6 were both among the top perturbed genes (**Figure 7J**). The accompanying MA plot further illustrated the global perturbation pattern following in silico Maf knockout (**Supplementary Figure 7D**). Together, these analyses support Maf as a candidate upstream regulator associated with the Mrc1^+^Gas6^+^ macrophage transcriptional program in AAA.

## Discussion

In the present study, we found that macrophage remodeling in AAA was dominated by a marked shift between two major macrophage programs. In the normal aorta, macrophages were mainly composed of Mrc1^+^Gas6^+^ and Cd36^+^Lpl^+^ subsets, whereas aneurysmal tissues became dominated by Thbs1^+^Spp1^+^ macrophages, accompanied by the emergence of a proliferative Cdca8^+^ subset (**Figures 1A, B**). Functionally, our data support the existence of four distinct macrophage states in AAA, with the most prominent shift occurring between an inflammatory Thbs1^+^Spp1^+^ program and an efferocytosis-associated/homeostatic Mrc1^+^Gas6^+^ program (**Figures 1D–I**, **2**, **3**, **6**).

AAA is widely recognized as an immune-driven vascular disease in which macrophages are central effectors of inflammatory remodeling, matrix destruction, and disease progression (1, 2, 4). This conceptual framework is consistent with the broader shift in cardiovascular macrophage biology away from the traditional M1/M2 paradigm and toward functionally defined macrophage states (8–10). Recent single-cell studies in AAA and related aortic disease have already identified substantial myeloid diversification, including interferon-inducible monocyte/macrophage populations in AAA and Il1rn^+^/Trem1^+^ macrophages in thoracic aortic aneurysm and dissection (12–14). In our dataset, a similarly high degree of macrophage diversification was also evident, with the most prominent remodeling occurring between Thbs1^+^Spp1^+^ and Mrc1^+^Gas6^+^ macrophages (**Figures 1A–C**). This pattern is conceptually aligned with a recent study of activated primary human macrophages, in which a pro-inflammatory subset displayed increased inflammatory chemokine expression and higher amino acid consumption, whereas a phagocytic subset exhibited enhanced phagocytic, efferocytotic, and chemotactic capabilities (19). Our Thbs1^+^Spp1^+^ macrophages shared several features with the former, including enrichment of inflammatory signaling, leukocyte chemotaxis, ROS-related programs, and amino acid biosynthesis/metabolism (**Figures 1D**, **2B, C**). By contrast, Mrc1^+^Gas6^+^ macrophages shared features with the latter, including enrichment of chemotactic and efferocytosis-related pathways (**Figures 3B, C**). At the same time, our data also suggest disease-specific features, because in AAA the Mrc1^+^Gas6^+^ subset was additionally characterized by increased apoptosis/p53-related signatures and progressive numerical decline (**Figures 1D–F**, **3D–J**).

The Thbs1^+^Spp1^+^ subset emerged as the dominant macrophage state in AAA and displayed a transcriptional profile consistent with inflammatory activation, chemotaxis, immune activation, oxidative stress, glycolytic remodeling, and mTORC1 signaling (**Figures 2A–E**). These features are compatible with previous studies showing that inflammatory macrophage states in aneurysmal disease are enriched for mediators such as Trem1 and related immune activation pathways (14). Our data further suggest that this subset is not simply inflammatory in an intrinsic sense, but also communication-active at the tissue level. CellChat analysis predicted SPP1 and THBS as major outgoing pathways from Thbs1^+^Spp1^+^ macrophages, with SMCs and ECs among the principal recipient populations (**Figures 2F, G**; **Supplementary Figures 2A, C**). This is consistent with prior computational analysis of AAA single-cell datasets highlighting SPP1 and THBS among altered communication pathways in aneurysmal tissues (20). Moreover, myeloid-derived thrombospondin-1 (TSP1/THBS1) has been reported to promote AAA pathogenesis, at least in part by suppressing TIMP1 expression and facilitating inflammatory macrophage infiltration (21). Together, these findings support the view that Thbs1^+^Spp1^+^ macrophages likely represent a major inflammatory communication state with prominent predicted communication potential in AAA.

By contrast, the Mrc1^+^Gas6^+^ subset appears to represent a macrophage program more closely linked to tissue homeostasis and apoptotic cell clearance. Several findings support its classification as an efferocytosis-associated state: this subset was enriched for Gas6 and Mertk, showed KEGG enrichment for efferocytosis-related pathways, and preferentially co-expressed Mrc1, Gas6, Mertk, Igf1, and Entpd1 (**Figures 3A–C**, **6B–D**). This is notable because the Gas6–MerTK axis is a well-established component of macrophage recognition and engulfment of apoptotic cells, and defective efferocytosis is now recognized as an important mechanism linking persistent inflammation to tissue injury in cardiovascular disease (11, 18). In atherosclerosis, failure of efferocytosis promotes apoptotic cell accumulation, secondary necrosis, and sustained lesional inflammation (11). In our study, the Mrc1^+^Gas6^+^ subset also showed enrichment of M2-associated markers, while M1-associated markers were enriched in the Thbs1^+^Spp1^+^ subset (**Figure 6A**), indicating partial correspondence with the classical M1/M2 framework. However, the combination of Gas6, Mertk, Igf1, and Entpd1, together with the enrichment of efferocytosis and chemotaxis pathways, suggests that the Mrc1^+^Gas6^+^ state is functionally more specific than a generic “M2-like” population (**Figures 3B, C**, **6B–D**).

Importantly, the decline of Mrc1^+^Gas6^+^ macrophages in AAA appeared to involve both numerical attrition and functional suppression. This subset was proportionally enriched in the normal aorta but significantly reduced in aneurysmal tissue (**Figures 1A, B**, **3J**). At the same time, it displayed increased apoptosis and p53-related activity (**Figures 1D–F**, **3D, E**), and TUNEL staining further showed increased death of MRC1^+^ cells in aneurysmal tissue (**Figure 3I**). In parallel, key molecules associated with apoptotic cell clearance and tissue homeostasis, including Gas6, Mertk, and Igf1, were diminished in AAA (**Figure 6D**). These findings suggest that AAA is associated not only with expansion of inflammatory macrophages, but also with loss of a macrophage subset that may contribute to local immune restraint and apoptotic cell clearance.

A major mechanistic insight from this study is the link between ERK activation and impairment of the Mrc1^+^Gas6^+^ program. ERK signaling has long been implicated in AAA, and earlier work showed that ERK activation is increased in aneurysmal tissues and that modulation of ERK-associated signaling can attenuate aneurysm formation (15, 16). Our data are consistent with that literature in showing increased p-ERK in aneurysmal aortic tissue (**Figure 6G**). More importantly, our results link ERK activation to the loss of Mrc1^+^Gas6^+^ macrophage-associated effector molecules. Under TNF-α-associated inflammatory stress, ERK inhibition increased Mertk, Gas6, and Igf1 expression in RAW264.7 and PMA-differentiated THP-1 macrophage-line models and enhanced macrophage uptake of apoptotic SMCs, whereas inhibition of MerTK suppressed this uptake (**Figures 6H–J**; **Supplementary Figures 6A, B**). This interpretation is also compatible with prior macrophage studies showing that efferocytosis- and resolution-related signaling is highly context dependent. For example, ERK5 activation has been linked to enhanced macrophage efferocytosis, whereas MerTK signaling promotes pro-resolving responses in macrophages (17, 18). Notably, Entpd1 did not change after ERK inhibition (**Figures 6H, I**; **Supplementary Figures 6A, B**), suggesting that the Mrc1^+^Gas6^+^ program is not uniformly regulated by ERK. Instead, ERK may preferentially influence the MerTK/Gas6/Igf1 arm of this program, whereas other components may be governed by distinct regulatory inputs.

Our data also point to MerTK as a likely key effector receptor within the efferocytosis-associated macrophage program in AAA. Among the three TAM receptors, only MerTK showed clear preferential enrichment in Mrc1^+^Gas6^+^ macrophages (**Figures 6B, D**). In functional assays, pharmacological inhibition of MerTK reduced macrophage efferocytosis-associated uptake (**Figure 6J**). Together with the enrichment of Gas6, these findings support the interpretation that the Gas6–MerTK axis is a major effector pathway associated with apoptotic cell clearance in this macrophage subset. This also fits with broader macrophage biology, in which MerTK is central to efferocytosis and inflammation resolution (11, 18).

Intercellular communication analysis further suggested that the inflammatory and efferocytosis-associated subsets may be embedded in distinct putative signaling modules. Thbs1^+^Spp1^+^ macrophages were predicted to act as the main senders of SPP1 and THBS signaling and preferentially targeted SMCs and ECs (**Figures 2F, G**; **Supplementary Figures 2A, C**). These pathways have been highlighted in prior AAA communication analyses, and THBS1 has also been functionally implicated in aneurysm progression (20, 21). By contrast, Mrc1^+^Gas6^+^ macrophages were characterized by CellChat-inferred outgoing CD39 and IGF signaling (**Figures 3F, G**; **Supplementary Figures 3A–C**). Because CD39 scavenges pro-inflammatory extracellular ATP and promotes the generation of immunosuppressive adenosine, thereby regulating T-cell activation and macrophage/T-cell responses (22, 23), the predicted Mrc1^+^Gas6^+^ towards T cell/Thbs1^+^Spp1^+^ macrophage signaling pattern may reflect a local immunoregulatory circuit. Likewise, the preferential targeting of ECs and SMCs by IGF signaling is compatible with prior work linking IGF-1 to macrophage–endothelial cell crosstalk and vascular cell survival-related responses (24). These communication data do not establish direct ligand-receptor activation or functional signaling, but they suggest that the two dominant macrophage subsets differ not only in intrinsic transcriptional state but also in the direction and biological context of their interactions with the surrounding aneurysmal niche.

Trajectory analysis further predicted that inflammatory and efferocytosis-associated macrophages occupied different branches of the macrophage trajectory based on inferred transcriptional-state, with Cdca8^+^ macrophages positioned at a plausible root state and terminal branches enriched either for inflammatory genes (Il1b, Trem1, Thbs1) or for homeostasis/efferocytosis-associated genes (Mrc1, Gas6, Igf1, Mertk) (**Figures 7A–E**; **Supplementary Figures 7A, B**). These findings suggest that AAA macrophages might diverge into distinct inflammatory and homeostatic/efferocytosis-associated state trajectories within the aneurysmal microenvironment. These findings should be interpreted as computational organization of macrophage transcriptional states rather than direct evidence of lineage progression or irreversible developmental divergence.

Within this framework, Maf emerged as a candidate upstream regulator associated with the Mrc1^+^Gas6^+^ macrophage program. SCENIC and pseudotime analyses associated Maf with the Mrc1^+^Gas6^+^ trajectory, whereas JASPAR motif prediction and in silico knockout supported its potential regulatory relationship with Mrc1^+^Gas6^+^-associated genes (**Figures 7F–J**; **Supplementary Figures 7A–D**). MAF-family transcription factors are established regulators of macrophage identity and adaptation. In particular, c-MAF has been shown to regulate perivascular macrophage identity, whereas MafB promotes anti-inflammatory macrophage polarization and cholesterol efflux (25–27). In our data, Maf showed expression dynamics similar to those of Mrc1, Gas6, and Igf1 (**Supplementary Figures 7A, B**), and in silico perturbation identified Mrc1 and Gas6 among the most affected genes after Maf knockout (**Figure 7J**; **Supplementary Figure 7D**). Together, these observations support Maf as a candidate regulator associated with the Mrc1^+^Gas6^+^ macrophage program in AAA.

Although the imbalance between Thbs1^+^Spp1^+^ and Mrc1^+^Gas6^+^ macrophages forms the central axis of this study, the other two subsets are also noteworthy. The Cdca8^+^ subset displayed a strong proliferative and cell-cycle-associated program (**Figures 4A–D**), suggesting the presence of a cycling macrophage state in AAA. The Cd36^+^Lpl^+^ subset is also of interest. This population exhibited stable enrichment of lipid transport, cholesterol handling, endocytosis, and PPAR-related programs (**Figures 5A–E**). These features are conceptually similar to lipid-associated or lipid-handling macrophage states described in atherosclerosis and metabolic disease (28, 29). In our dataset, this subset also preferentially received CD200 signaling (**Figures 5F, G**; **Supplementary Figures 5A–C**), an observation compatible with prior work showing that the CD200–CD200R axis restrains lesional macrophage activation and monocyte recruitment in atherosclerosis (30).

Several limitations of this study should be acknowledged. First, the *in vivo* analysis was performed in the peri-adventitial elastase model, and it remains uncertain to what extent the same macrophage-state architecture is conserved across other aneurysm models or in human AAA. Second, the scRNA-seq discovery dataset contained one pooled library per condition. This design increases the number of cells captured from small murine infrarenal aortas but does not provide independent sequencing-level biological replicates and therefore cannot support formal statistical testing of macrophage subset proportions across groups. Third, although our findings suggest that Mrc1^+^Gas6^+^ macrophages are efferocytosis-associated, *in vivo* apoptotic cell clearance by this subset was not directly traced within the aneurysmal aorta. Fourth, the mechanistic evidence linking ERK to this macrophage program currently relies on pharmacological perturbation in macrophage-line models. However, macrophage-line models cannot fully recapitulate primary aortic MRC1^+^GAS6^+^ macrophages *in vivo*. TNF-α stimulation improves disease relevance by modeling one inflammatory cue present in AAA, but it still does not reproduce the full complexity of the aneurysmal microenvironment. Future studies using primary macrophages, sorted aortic macrophage subsets, or macrophage-specific ERK modulation *in vivo* will be required to establish this mechanism more directly. Fifth, flow cytometry-based quantification of F4/80^+^PKH26^+^ events cannot by itself completely distinguish internalized apoptotic material from surface-bound fragments or membrane dye transfer. Sixth, Maf remains a candidate regulator and still requires direct mechanistic validation. Finally, pseudotime analysis was used only to infer macrophage transcriptional-state organization. Because its directionality depends partly on root-state assignment, the selection of Cdca8^+^ macrophages as a computational root does not establish them as the biological origin of other macrophage states. Lineage-tracing or fate-mapping studies will be needed to validate whether these inferred relationships reflect true developmental transitions *in vivo*.

Despite these limitations, our findings provide a more selective framework for interpreting macrophage dysfunction in AAA. Rather than viewing aneurysmal inflammation solely as excessive accumulation of inflammatory macrophages, our data suggest that disease progression may also reflect failure to preserve a macrophage program associated with apoptotic cell clearance and local immune restraint (**Figures 3**, **6**, **7**). Future work should determine whether restoring efferocytosis-associated macrophage function—either by preserving Mrc1^+^Gas6^+^ macrophages or by targeting pathways associated with their dysfunction, such as ERK-related signaling and transcriptional regulators including Maf—can attenuate aneurysm progression. More broadly, these data support the idea that rebalancing inflammatory and pro-resolution macrophage programs may represent a more promising direction for AAA intervention than non-selective suppression of macrophage accumulation.

## Methods

### Animals

Male C57BL/6J mice (10–12 weeks old, 18–25 g; SPF (Beijing) Biotechnology Co., Ltd., China) were housed in a specific pathogen-free facility under controlled conditions with a 12-hour light/dark cycle and provided with standard chow and water ad libitum. Mice were selected based on predefined inclusion criteria of general health and physiological status. Exclusion criteria included marked illness, abnormal body weight, or unexpected death during the experimental period. Animals were monitored daily for signs of pain or distress. Humane endpoints included >20% body weight loss, severe lethargy, abnormal posture, or ulceration. Mice meeting humane endpoint criteria were euthanized under deep isoflurane anesthesia followed by cardiac puncture and cervical dislocation. All animal experiments were approved by the Ethics Review Board of Peking Union Medical College Hospital (Approval No. XHDW-2023-083-2) and conducted in accordance with NIH guidelines for the care and use of laboratory animals.

### Elastase-induced AAA model

The elastase-induced AAA model was established as previously described. Briefly, 10–12-week-old male C57BL/6J mice were anesthetized by intraperitoneal injection of 1.25% tribromoethanol (0.02 mL/g body weight). After confirmation of adequate anesthesia, mice were placed on a thermostatically controlled surgical platform to maintain core temperature at 37 °C. Under sterile conditions, a midline laparotomy was performed to expose the infrarenal abdominal aorta between the left renal artery and the iliac bifurcation.

A sterile cotton pad soaked with 30 μL porcine pancreatic elastase (Type I, 20 U/mL; Sigma-Aldrich, Cat. No. E1250) was wrapped around the isolated aortic segment and incubated *in situ* for 30 min. In Sham-operated mice, heat-inactivated elastase (100 °C for 20 min) was applied in an identical manner. After incubation, the cotton pad was removed and the abdominal cavity was rinsed twice with sterile saline to remove residual elastase. The abdominal wall and skin were closed in layers. Topical 1% xylocaine and penicillin were applied to the incision site, and routine postoperative analgesic and antibiotic care were provided. Mice were monitored until full recovery and maintained for 7 or 14 days according to the experimental design (Sham, Elastase 7d, and Elastase 14d). At the indicated endpoints, mice were euthanized under deep anesthesia, and the infrarenal abdominal aorta was exposed under a stereomicroscope before tissue collection. The maximal external diameter of the elastase-treated infrarenal aortic segment was measured using a digital caliper under a stereomicroscope. After diameter measurement, aortic tissues were harvested for downstream analyses.

### Tissue dissociation and single-cell RNA sequencing

Abdominal aortas were harvested from mice in each experimental group and immediately perfused with ice-cold phosphate-buffered saline (PBS) to remove residual blood. Perivascular adipose tissue and intraluminal thrombus were carefully removed under a stereomicroscope. For all experiments, the infrarenal abdominal aorta, extending from below the renal arteries to the iliac bifurcation, was collected.

Infrarenal abdominal aortas from five mice per condition were pooled to generate one single-cell suspension per group. Pooled aortic tissues were minced into small fragments and digested at 37 °C for 1.5 h in 1 mL enzyme solution containing 450 U/mL collagenase type I (Gibco, Cat. No. 17100-017), 125 U/mL collagenase type XI (Sigma, Cat. No. C7657), 60 U/mL hyaluronidase type I-S (Sigma, Cat. No. H3506), and 60 U/mL DNase I (Sigma, Cat. No. DN25) in sterile PBS. Gentle agitation was applied during digestion, and the digestion step was repeated if necessary to ensure adequate tissue dissociation. The resulting single-cell suspensions were filtered through a 70-μm cell strainer, centrifuged at 300 × g for 5 min, and resuspended in Opti-MEM (Gibco) supplemented with 10% fetal bovine serum (FBS). Cell viability was assessed by trypan blue exclusion, and only samples with viability >70% were used for downstream library preparation.

Single-cell RNA-seq libraries were generated using a droplet-based microfluidic platform (Chromium Controller, 10x Genomics) at the Single Cell Core Facility of OE Biotech Co., Ltd. (Shanghai, China). Libraries were prepared using the Chromium Single Cell 3′ Library & Gel Bead Kit v3 (10x Genomics, PN-1000075) according to the manufacturer’s protocol. Approximately 2,500 cells were targeted per library, with an expected sequencing depth of ~200 million reads per sample. Twelve cycles were used for cDNA amplification and twelve cycles for sample index PCR. Library quality was assessed using an Agilent 2100 Bioanalyzer before sequencing. Sequencing was performed on an Illumina NovaSeq 6000 platform (read 1 = 28 bp, index = 8 bp, read 2 = 150 bp). The tissue dissociation and sequencing workflow followed the same experimental framework as in the previous manuscript.

### scRNA-seq data processing and analysis

Raw sequencing data were processed using Cell Ranger (version 7.1.0, 10x Genomics). FASTQ files were generated using the mkfastq module, and reads were aligned to the mouse reference genome (mm10) using the count function to obtain gene-by-cell expression matrices. To reduce ambient RNA contamination and background noise inherent to droplet-based scRNA-seq, CellBender (version 0.3.0) was applied using the remove-background function with default parameters.

Downstream analyses were performed in R (version 4.3.1) using Seurat (version 5.0.3). Cells with fewer than 200 detected genes, more than 7,500 detected genes, or more than 10% mitochondrial transcripts were excluded. The lower gene-count threshold was used to remove low-complexity droplets, whereas the mitochondrial threshold was used to reduce stressed or damaged cells. The upper threshold of 7,500 detected genes was used as a conservative filter for droplets with abnormally high gene counts, which may represent doublets or multiplets. In addition to threshold-based QC, doublet detection was performed separately for each library using DoubletFinder (version 2.0.4). Predicted doublets were removed before normalization, Harmony integration, clustering, and downstream analyses. After QC and DoubletFinder-based doublet removal, a total of 1,394 cells from Sham, 1,801 cells from Elastase 7d, and 1,411 cells from Elastase 14d were retained for integrated analysis.

Following normalization, 2,000 highly variable genes were identified using the “vst” method, and the data were scaled before principal component analysis (PCA). For integrated analysis of AAA progression, datasets from the Sham, Elastase 7d, and Elastase 14d groups were combined, and batch effects were corrected using Harmony. Dimensionality reduction, clustering, and visualization were performed based on Harmony-corrected principal components. In parallel, unintegrated analyses were also examined when preservation of condition-specific transcriptional programs was required.

Graph-based clustering was performed using a shared nearest neighbor (SNN) approach implemented in Seurat, and clusters were visualized by Uniform Manifold Approximation and Projection (UMAP). Marker genes for each cluster were identified using the Wilcoxon rank-sum test with thresholds of log2 fold change > 0.25 and adjusted P < 0.05. Major aortic cell populations were annotated using canonical marker genes. For macrophage-focused analyses, macrophage clusters were extracted from the integrated dataset and re-clustered. Gene expression patterns were visualized using FeaturePlot, VlnPlot, DotPlot, and DoHeatmap functions in Seurat.

### Functional enrichment analysis

Functional enrichment analysis was performed using the clusterProfiler R package. Differentially expressed genes and cluster-specific marker genes were converted to Entrez IDs using the org.Mm.eg.db annotation database. Gene Ontology (GO) enrichment was conducted for the Biological Process (BP) ontology using the enrichGO function with a Benjamini–Hochberg adjusted P value cutoff of 0.05. Redundant terms were filtered, and the top enriched categories were visualized using dot plots.

In GO dot plots, the gene_ratio represents the proportion of input genes annotated to a given GO term, calculated as Count divided by the total number of input genes analyzed. The term_ratio was defined as Count divided by the number of background genes annotated to that GO term, reflecting the density of enrichment relative to the size of the term itself.

To visualize representative GO terms together with their core driving genes, Sankey diagrams were generated using the sankeyGoPlot function from the GseaVis package. For each macrophage subset, the top enriched GO terms and their leading-edge genes were displayed as gene–term bipartite networks.

KEGG pathway enrichment was also performed using clusterProfiler. In KEGG dot plots, point size indicates gene count and color indicates –log10(q value).

### Rank-based gene set scoring with irGSEA

Rank-based single-cell gene set enrichment analysis was performed using the irGSEA package. Hallmark gene sets from MSigDB were used to evaluate pathway activities across macrophage subsets. The irGSEA.score() function was applied to the Seurat object using default parameters unless otherwise specified. AUCell, UCell, and ssGSEA were applied in parallel when required for cross-method comparison.

### Single-cell pathway analysis using escape and PROGENy

Hallmark gene sets for Mus musculus were obtained from the msigdbr package and formatted as gene-set lists. Enrichment scores were calculated using the runEscape function with the AUCell method, and the resulting scores were stored as a new assay for downstream heatmap and group-comparison analyses.

Pathway activities were inferred using the progeny package with organism = “Mouse”, top = 500, perm = 1, and return_assay = TRUE. The resulting PROGENy assay was scaled prior to visualization and summarization across macrophage subsets.

### Cell-cell communication analysis

Cell-cell communication analysis was performed using CellChat (version 1.6.1) to infer putative ligand-receptor communication patterns in the murine aortic microenvironment. Normalized expression matrices and Seurat-derived cell-type annotations were used as input. Separate CellChat analyses were performed for each group (Sham, Elastase 7d, and Elastase 14d), and merged analyses were generated when direct group comparison was required.

Ligand-receptor interactions were predicted using the curated mouse interaction database provided by CellChat. Network-level metrics, including inferred interaction number, interaction strength, signaling role, and relative information flow, were calculated and compared across groups. In macrophage-subset analyses, global incoming and outgoing interaction patterns were first examined for each subset, after which subset-relevant signaling pathways were selected for focused visualization using signaling network plots, bubble plots of ligand-receptor pairs, and pathway-specific ligand-receptor contribution plots.

### Trajectory analysis

Trajectory analysis of macrophages was performed using Monocle2 (version 2.30.1) to explore transcriptional-state organization within the macrophage compartment. Raw UMI count matrices were extracted from Seurat objects and converted into a CellDataSet. Genes expressed in fewer than 10 cells were excluded, and size factors and dispersion estimates were computed using the default negative binomial model.

Highly variable genes identified in Seurat were first used as candidate genes. Differential expression across macrophage subsets was then tested in Monocle2, and genes with q < 0.01 were used as ordering genes for trajectory reconstruction. Dimensionality reduction was performed using the DDRTree algorithm, and cells were ordered in pseudotime with manual specification of the root state according to biologically inferred origin.

Key gene-expression dynamics along pseudotime were visualized on the trajectory plot. Branch-dependent gene regulation at the major bifurcation point was analyzed using Branched Expression Analysis Modeling (BEAM), and branch-specific heatmaps were generated for to visualize pseudotime-associated transcriptional patterns.

### CytoTRACE2 analysis

To estimate the relative developmental potential of macrophage subsets, CytoTRACE2 scores were calculated from the macrophage expression matrix using the CytoTRACE2 algorithm (31). Higher CytoTRACE2 scores were interpreted as greater inferred developmental potential. Subset-level CytoTRACE2 distributions were compared across macrophage clusters and were used to guide root-state assignment for trajectory inference.

### Transcription factor regulatory network analysis

Transcription factor regulatory network inference was performed using the SCENIC workflow on macrophages extracted from the integrated single-cell dataset. Gene regulatory networks were inferred using GENIE3. Co-expression modules were subsequently refined into regulons using motif enrichment analysis with RcisTarget and curated mouse motif databases. AUCell was used to calculate regulon activity scores for each cell.

To identify subset-enriched transcription factors, regulon specificity scores (RSS) were computed from the AUCell-derived regulon activity matrix and macrophage subset annotations. Regulons with high RSS values were visualized as heatmaps and ranked to identify subset-specific candidate regulators. Regulon-target relationships were further filtered based on differential expression and Spearman correlation and visualized as networks.

### *In silico* prediction of Maf binding sites using JASPAR

Putative Maf-binding sites were predicted using JASPAR (32). The Maf DNA-binding motif retrieved from JASPAR was used to scan the 2-kb upstream promoter regions of Mrc1, Gas6, Mertk, and Igf1. Sites with a relative profile score of ≥70% were considered candidate Maf-binding sites, and the highest-scoring predicted motif for each gene was selected for visualization.

### Virtual knockout analysis

Virtual knockout analysis was performed using the scTenifoldknk package in R with default parameters. Briefly, the macrophage scRNA-seq expression matrix was used to construct a wild-type single-cell regulatory network, after which Maf was computationally deleted in silico. Gene perturbation was quantified by comparing the perturbed and unperturbed manifolds, and genes were ranked according to perturbation magnitude. Top perturbed genes were visualized in bar plots and MA plots.

### Cell culture and treatment

RAW264.7 murine macrophages were cultured in DMEM, high glucose (Gibco, Cat. No. 11965092) supplemented with 10% fetal bovine serum (Gibco, Cat. No.10099141C) and 1% penicillin-streptomycin (Sigma, Cat. No. V900929) at 37 °C in a humidified incubator containing 5% CO2. Smooth muscle cells (SMCs) used in the efferocytosis assay were maintained in complete medium containing 10% fetal bovine serum under standard culture conditions.

THP-1 human monocytic cells were cultured in RPMI-1640 medium (Gibco, Cat. No. C11875500BT) supplemented with 10% fetal bovine serum (Gibco, Cat. No.10099141C) and 1% penicillin-streptomycin (Sigma, Cat. No. V900929) in a humidified incubator containing 5% CO2. To induce macrophage differentiation, THP-1 cells were treated with phorbol 12-myristate 13-acetate (PMA) (MCE, Cat. No. HY-18739) at a final concentration of 100 ng/mL for 48h, followed by resting in PMA-free complete medium for 24h before subsequent stimulation and treatment.

For inflammatory stimulation, RAW264.7 macrophages and PMA-differentiated THP-1 macrophages were treated with recombinant TNF-α at a final concentration of 20 ng/mL for 6h. For ERK inhibition experiments, cells were treated with U0126 (MCE, Cat. No. HY-12031A) at a final concentration of 10 μM for 24 h. For MerTK inhibition experiments, RAW264.7 cells were treated with UNC2025 (MCE, Cat. No. HY-12344) at a final concentration of 10 nM for 4 h. Vehicle-treated cells served as controls.

### Quantitative real-time PCR

Total RNA was extracted from abdominal aortic tissues or cultured cells using TRIzol reagent (Invitrogen, Cat. No. 15596018) according to the manufacturer’s instructions. RNA concentration and purity were determined using a NanoDrop spectrophotometer. Complementary DNA was synthesized using PrimeScript RT Reagent Kit with gDNA Eraser (TaKaRa, Cat. No. RR047A). Quantitative PCR was performed using TB Green Premix Ex Taq II (Tli RNaseH Plus) (TaKaRa, Cat. No. RR820A) on a QuantStudio 5 Real-Time PCR System (Thermo Fisher Scientific). Gene expression levels were normalized to *Gapdh*, and relative transcript levels were calculated using the 2^-ΔΔCt method. Three biologically independent samples were analyzed per group, and each reaction was run in technical triplicate unless otherwise specified.

### Western blot analysis

Protein lysates were extracted from abdominal aortic tissues or cultured cells using RIPA lysis buffer supplemented with protease and phosphatase inhibitor cocktail. Protein concentrations were determined using a BCA protein assay. Equal amounts of protein (20-30 μg) were separated by SDS-PAGE and transferred to PVDF membranes. After blocking with 5% nonfat milk in TBST for 1 h at room temperature, membranes were incubated overnight at 4 °C with primary antibodies according to the manufacturers’ recommended dilutions. Primary antibodies used in this study included total-ERK1/2 (t-ERK1/2), phospho-ERK1/2 (p-ERK1/2), MERTK, ENTPD1/CD39, GAS6, IGF1, and GAPDH. After washing, membranes were incubated with HRP-conjugated secondary antibodies and visualized using enhanced chemiluminescence. Images were acquired using a chemiluminescence imaging system, and densitometric quantification was performed using ImageJ software (version 1.52p). For ERK phosphorylation analysis, the grayscale intensities of the p-ERK1 and p-ERK2 bands were summed and normalized to the summed intensity of total ERK1 and ERK2 from the corresponding sample. For other target proteins, band intensities were normalized to GAPDH. Comparisons were restricted to samples run on the same gel and processed under identical exposure conditions.

### Induction of apoptotic smooth muscle cells

SMCs were labeled with PKH26 Red Cell Membrane Staining Kit (Solarbio, Cat. No. D0030) according to the manufacturer’s instructions and washed thoroughly to remove unbound dye. After labeling, SMCs were seeded and allowed to adhere for 12 h. Apoptosis was then induced using the Apoptosis Inducer Kit (TNF-α + SM-164) (Beyotime, Cat. No. C0006S) by adding the apoptosis inducer directly to the culture medium for 4 h. Before co-culture with macrophages, apoptosis induction was validated by Annexin V/PI flow cytometry according to the manufacturer’s protocol (Yeasen, Cat. No. 40302ES50). Annexin V-positive and PI-negative cells were considered apoptotic, and the proportion of apoptotic SMCs was quantified before use in the efferocytosis assay. After induction, apoptotic PKH26-labeled SMCs were collected and used for co-culture with RAW264.7 macrophages.

### Macrophage efferocytosis assay

For efferocytosis assays, apoptotic PKH26-labeled SMCs were added to RAW264.7 macrophages at an apoptotic SMC-to-macrophage ratio of 3:1 and co-cultured for 12 h. Where indicated, macrophages were pretreated with U0126 or UNC2025 before co-culture. Cytochalasin D was used as an actin-dependent phagocytosis-inhibition control. For the phagocytosis-inhibition control, macrophages were pretreated with cytochalasin D (5 μM; MCE, Cat. No. HY-N6682) for 30 min before co-culture with PKH26-labeled apoptotic SMCs. At the end of co-culture, cells were harvested and stained with anti-F4/80 antibody to identify macrophages. Flow cytometry was used to quantify the proportion of F4/80-positive PKH26-positive cells, which was interpreted as macrophages associated with apoptotic SMC-derived PKH26 signal.

### Histology, immunofluorescence staining, and TUNEL assay

For immunofluorescence staining, paraffin-embedded abdominal aortic sections (4 μm) were deparaffinized, rehydrated, and subjected to heat-induced antigen retrieval using EDTA buffer (ZSGB-BIO, Cat. No. ZLI-9072). After blocking with 5% normal goat serum, sections were incubated overnight at 4 °C with primary antibodies according to the manufacturers’ recommended dilutions. Primary antibodies used in this study included CD68, SPP1, MRC1/CD206, CDCA8, MERTK, ENTPD1/CD39, GAS6, and IGF1. Fluorescent secondary antibodies or directly labeled primary antibodies were then applied, followed by DAPI counterstaining. For selected markers, TSA-based multiplex immunofluorescence was performed using the TSA Fluorescence Kit (Panovue, Cat# 10268100050) according to the manufacturer’s protocol. Images were acquired using a Zeiss Axio Imager Z2 fluorescence microscope under identical acquisition settings for matched comparisons. For selected images, line-scan analysis was performed in ImageJ (version 1.52p) to quantify fluorescence intensity of the indicated channels along the selected line segment.

For TUNEL assays, apoptosis was detected using the One Step TUNEL Apoptosis Assay Kit (Beyotime, Cat. No. C1089). In combined TUNEL/immunofluorescence experiments, sections were first stained for MRC1 by standard immunofluorescence, including incubation with fluorescent secondary antibody, and were then subjected to TUNEL staining according to the manufacturer’s protocol.

### Flow cytometry

Single-cell suspensions from murine infrarenal abdominal aortas were prepared as described above and filtered through a 70-μm cell strainer. Cells were stained with fluorophore-conjugated antibodies against CD45, CD11b, and F4/80 to identify macrophages. For detection of subset-associated markers, SPP1 and MRC1 were analyzed in separate staining panels. For intracellular detection of SPP1, cells were fixed and permeabilized before SPP1 staining. Data were acquired on an LSRFortessa flow cytometer and analyzed using FlowJo X 10.0.7r2.

Unstained and single-stained controls were used for fluorescence compensation. Fluorescence minus one (FMO) controls were performed for the target-marker channels, SPP1 and MRC1. For the SPP1 FMO control, all antibodies in the SPP1 staining panel were included except the anti-SPP1 antibody. For the MRC1 FMO control, all antibodies in the MRC1 staining panel were included except the anti-MRC1 antibody. These FMO controls were used to define the background fluorescence and positivity gates for SPP1^+^ and MRC1^+^ macrophages, and the same gates were applied consistently across all samples within the same staining batch.

For aortic tissue validation, the gating strategy was as follows. First, the major intact cell population was selected on an FSC-A versus SSC-A plot to exclude debris and very small particles. Singlets were then gated using FSC-A versus FSC-H. Within the singlet population, CD45^+^CD11b^+^ myeloid cells were selected, and macrophages were further defined as CD45^+^CD11b^+^F4/80^+^ cells. The percentages of SPP1^+^ and MRC1^+^ cells were quantified within the CD45^+^CD11b^+^F4/80^+^ macrophage gate.

For the efferocytosis assay, co-cultured cells were harvested and stained with anti-F4/80 antibody. After debris exclusion and singlet gating, F4/80^+^ macrophages were selected, and PKH26^+^ events within the F4/80^+^ macrophage gate were quantified as macrophages associated with PKH26-labeled apoptotic SMCs.

A dedicated viability dye was not included in these flow cytometry panels. Therefore, debris and abnormal events were excluded by FSC/SSC and singlet gating, but dye-based viability discrimination was not performed.

### Statistical analysis

Data are presented as mean ± SEM for normally distributed variables, as indicated in the corresponding figure legends. Normality was assessed using the Shapiro-Wilk test, and homogeneity of variance was evaluated before parametric testing. For comparisons between two groups, an unpaired two-tailed Student’s t test was used for normally distributed data with equal variances. For comparisons involving more than two groups, one-way ANOVA followed by Tukey’s *post hoc* test was used for data with homogeneous variances. For scRNA-seq analyses, differential gene expression between cell clusters or experimental groups was assessed using the Wilcoxon rank-sum test, and multiple testing was corrected using the Benjamini–Hochberg method. All statistical analyses and graphical visualizations were performed using GraphPad Prism (version 10.0), and R (version 4.3.1). A two-tailed P value < 0.05 or adjusted P value < 0.05 was considered statistically significant.

## Conclusions

In summary, our study identifies a macrophage state imbalance in AAA characterized by expansion of Thbs1^+^Spp1^+^ inflammatory macrophages and attrition of Mrc1^+^Gas6^+^ efferocytosis-associated macrophages. ERK overactivation is associated with suppression of Mrc1^+^Gas6^+^ macrophage effectors and impaired efferocytotic function, whereas Maf emerges as a candidate regulator linked to this protective program. These findings provide a framework for understanding defective macrophage-mediated resolution in AAA and suggest that preserving or restoring efferocytosis-associated macrophage function may represent a potential therapeutic direction in aneurysmal disease.

## Data Availability

The data presented in the study are deposited in the Genome Sequence Archive (GSA) repository, accession number CRA045012.
