# Supplementary material for: Efferocytosis-associated Mrc1+Gas6+ macrophages are linked to abdominal aortic aneurysm progression through ERK-associated dysfunction
Source: Front Immunol. 2026 Jul 7;17:1863507. doi: 10.3389/fimmu.2026.1863507 (PMC13384856; doi:10.3389/fimmu.2026.1863507)
Supplement: Supplementary file 1 [file Supplementaryfile1.docx]

Supplementary Material

# Supplementary Figures and Tables

## Supplementary Figures


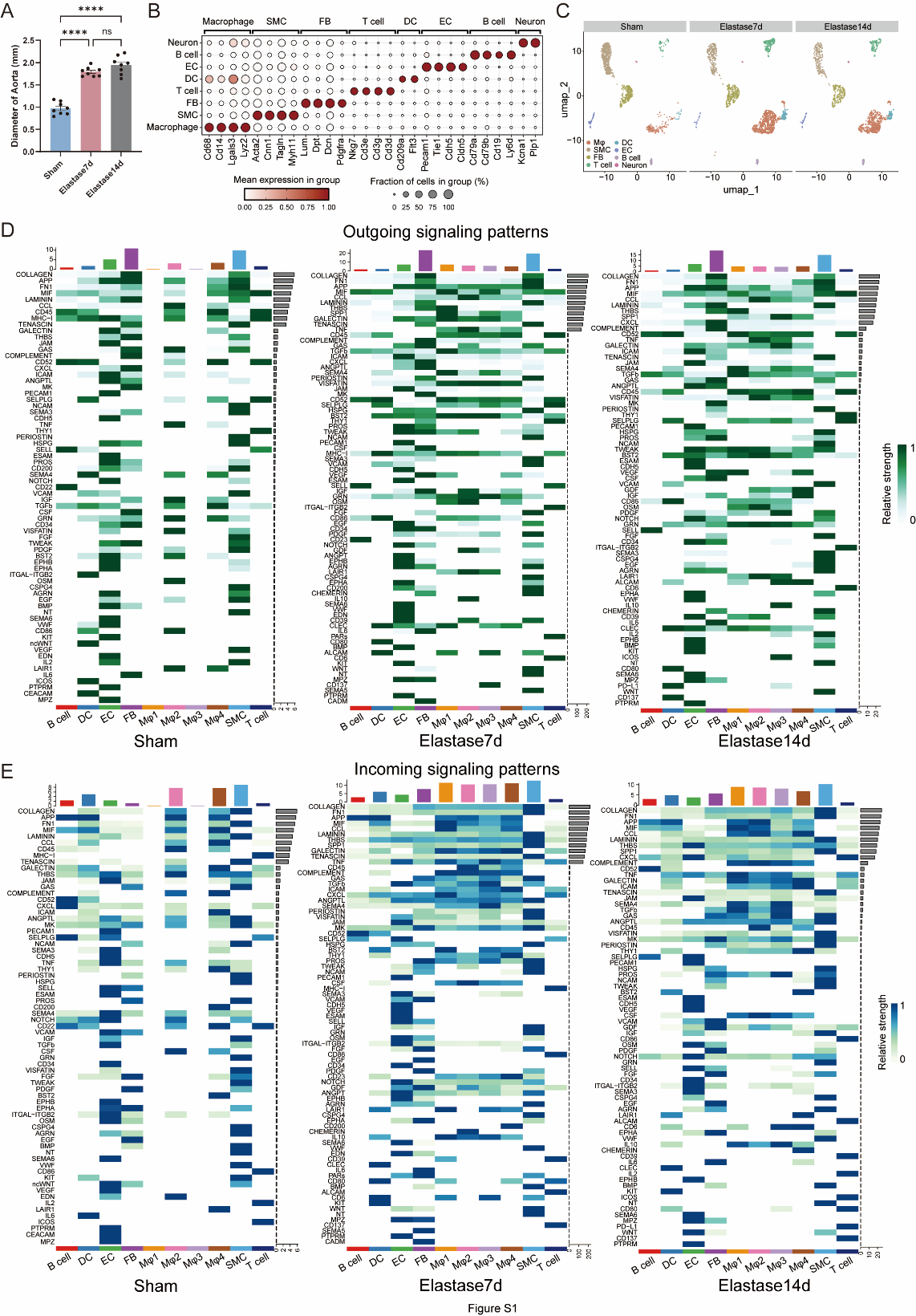


**Supplementary Figure 1. Global cellular landscape and cell-cell communication patterns in elastase-induced AAA.**

Mice were grouped into Sham, Elastase 7d, and Elastase 14d. For each group, cells from infrarenal abdominal aortas were pooled from 5 mice.

A, Box plot showing maximal external diameter of the infrarenal abdominal aorta in Sham, Elastase 7d, and Elastase 14d mice.

B, Dot plot showing canonical marker genes used for cell-type annotation, including macrophages (Mφ), smooth muscle cells (SMCs), fibroblasts (FBs), T cells, dendritic cells (DCs), endothelial cells (ECs), B cells, and neurons. Dot size indicates the fraction of cells expressing the indicated gene, and color indicates the mean expression level.

C, UMAPs of major aortic cell populations stratified by group.

D, Heatmaps showing outgoing signaling patterns inferred by CellChat across major cell populations in Sham, Elastase 7d, and Elastase 14d groups. Color intensity indicates relative signaling strength.

E, Heatmaps showing incoming signaling patterns inferred by CellChat across major cell populations in Sham, Elastase 7d, and Elastase 14d groups. Color intensity indicates relative signaling strength.

Data are presented as mean ± SEM (A). Statistical significance was determined using one-way ANOVA with Tukey’s post hoc test (A). ****P ≤ 0.0001; ns, not significant.


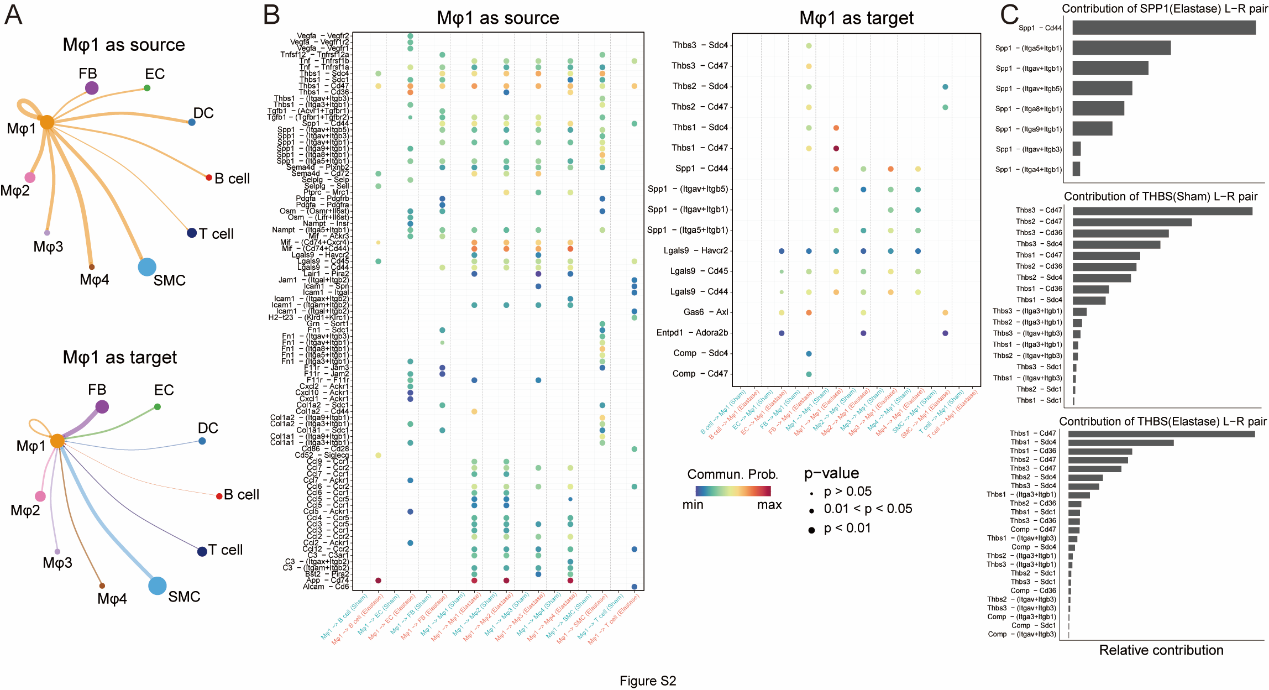


**Supplementary Figure 2. CellChat analysis of Thbs1^+^Spp1^+^ macrophage-associated intercellular communication in AAA.**

A, CellChat-inferred interaction patterns showing Mφ1 as a putative signaling sender (upper panel) and receiver (lower panel).

B, Bubble plots of ligand-receptor pairs associated with Mφ1 outgoing signaling (left) and incoming signaling (right) in Sham and Elastase. Dot size indicates p-value, and color reflects inferred communication probability.

C, Relative contribution of individual ligand-receptor pairs within the SPP1 and THBS signaling pathways in Sham and Elastase.


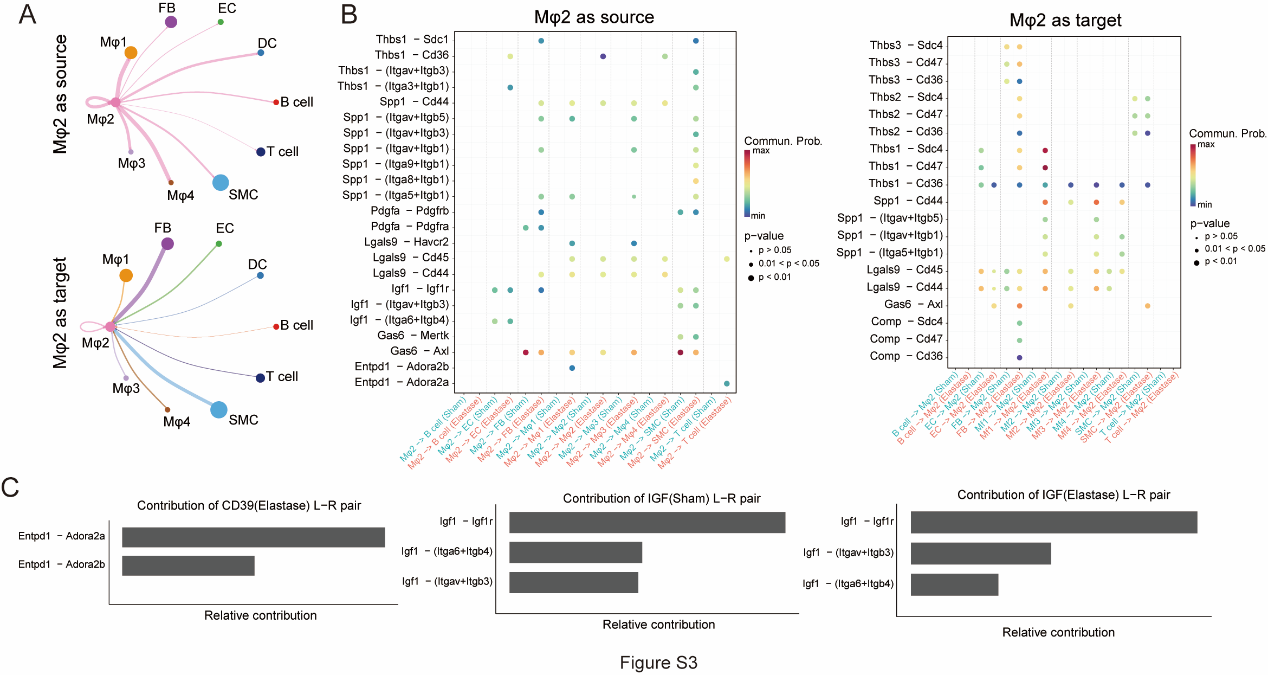


**Supplementary Figure 3. CellChat analysis of Mrc1^+^Gas6^+^ macrophage-associated intercellular communication in AAA.**

A, CellChat-inferred interaction patterns showing Mφ2 as a putative signaling sender (upper panel) and receiver (lower panel) in Sham and Elastase.

B, Bubble plots of ligand-receptor pairs associated with Mφ2 outgoing signaling (left) and incoming signaling (right) in Sham and Elastase. Dot size indicates p-value, and color reflects inferred communication probability.

C, Relative contribution of individual ligand-receptor pairs within the CD39 and IGF signaling pathways in Sham and Elastase.


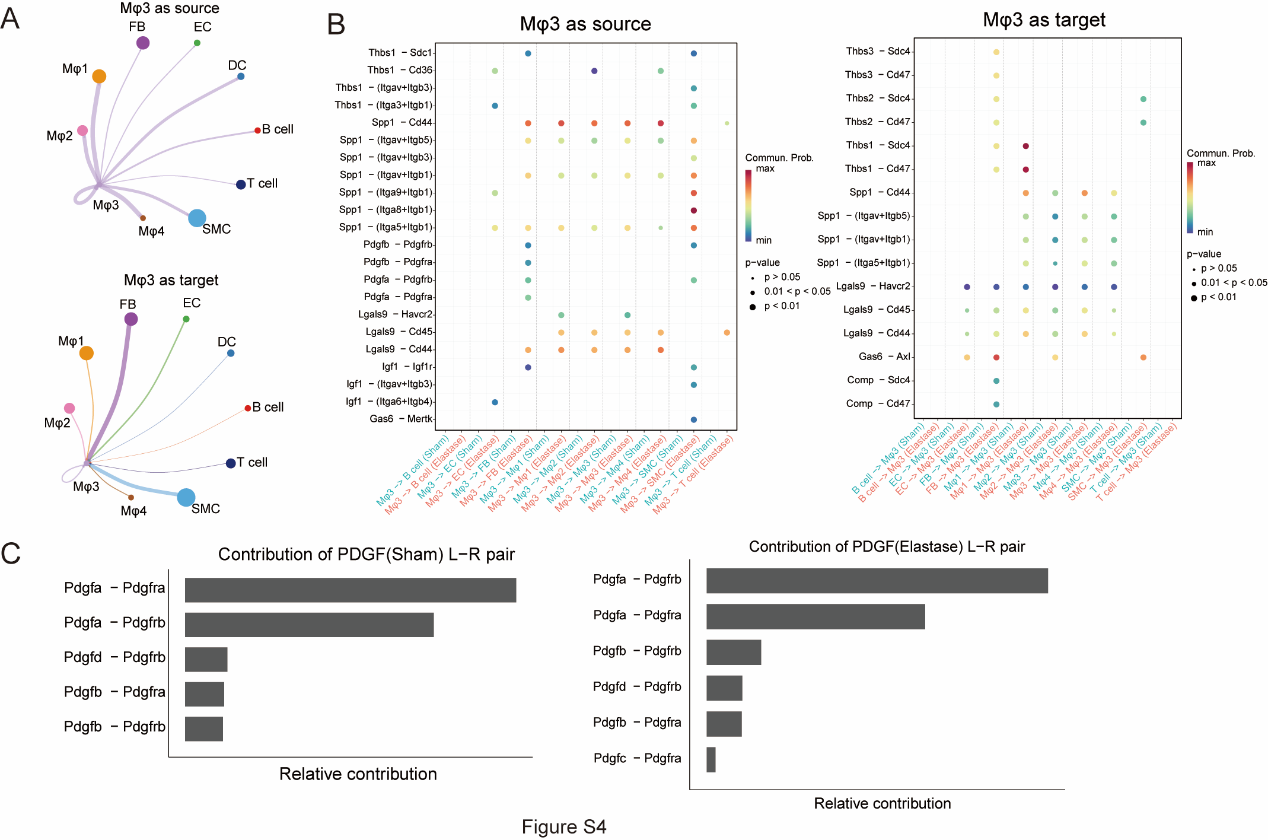


**Supplementary Figure 4. CellChat analysis of Cdca8^+^ macrophage-associated intercellular communication in AAA.**

A, CellChat-inferred interaction patterns showing Cdca8^+^ macrophages as a putative signaling sender (upper panel) and receiver (lower panel) in Sham and Elastase samples.

B, Bubble plots of ligand-receptor pairs associated with Cdca8^+^ macrophage outgoing signaling (left) and incoming signaling (right) in Sham and Elastase. Dot size indicates p-value, and color reflects inferred communication probability.

C, Relative contribution of individual ligand-receptor pairs within the PDGF signaling pathway in Sham and Elastase.


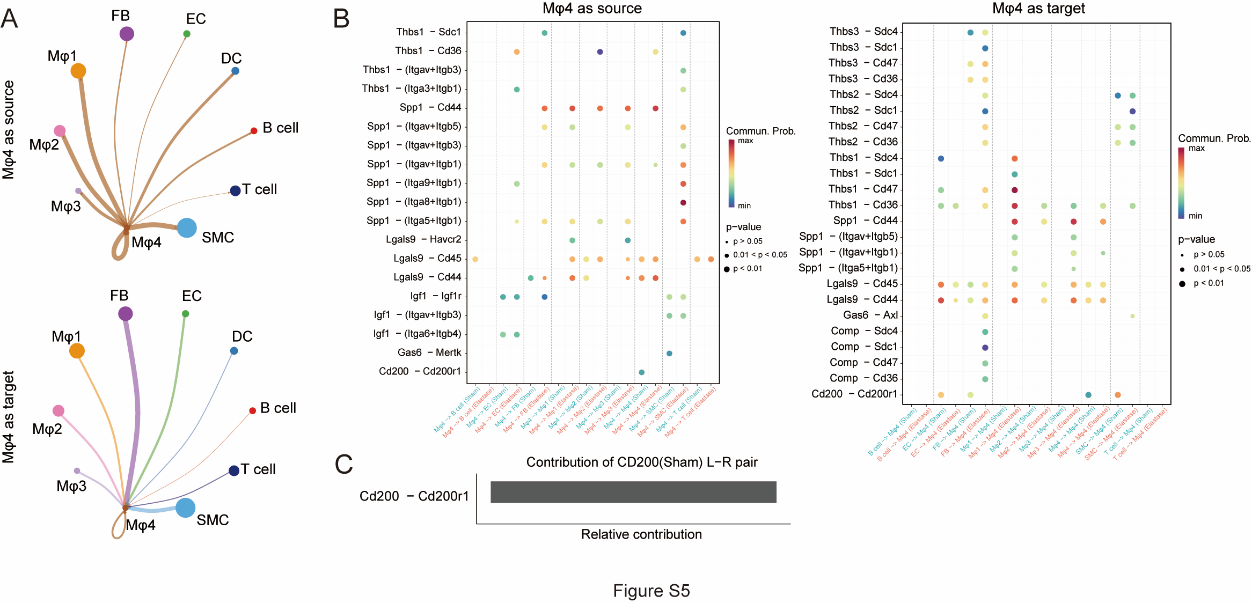


**Supplementary Figure 5. CellChat analysis of Cd36^+^Lpl^+^ macrophage-associated intercellular communication in AAA.**

A, CellChat-inferred interaction patterns showing Cd36^+^Lpl^+^ macrophages as a signaling putative sender (upper panel) and receiver (lower panel) in Sham and Elastase samples.

B, Bubble plots of ligand-receptor pairs associated with Cd36^+^Lpl^+^ macrophage outgoing signaling (left) and incoming signaling (right) in Sham and Elastase. Dot size indicates p-value, and color reflects inferred communication probability.

C, Relative contribution of individual ligand-receptor pairs within the CD200 signaling pathway in Sham.


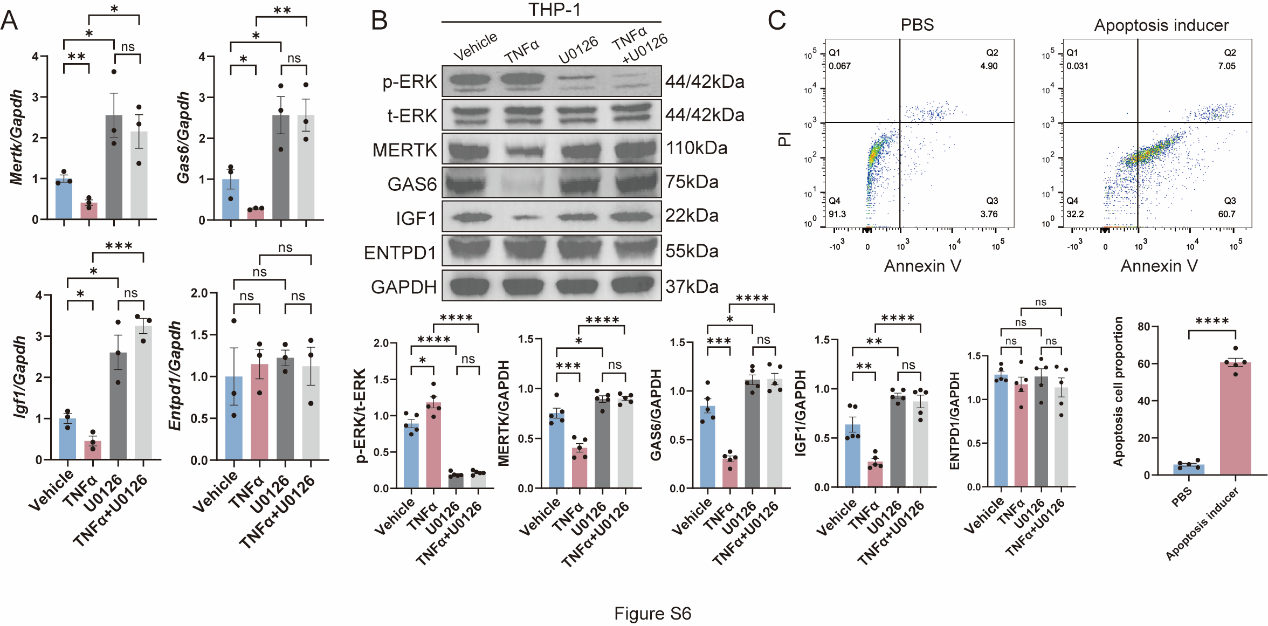


**Supplementary Figure 6. ERK inhibition restores MERTK/GAS6/IGF1 expression in THP-1-derived macrophages and validation of SMC apoptosis before the efferocytosis assay.**

A, qPCR analysis of MERTK, GAS6, IGF1, and ENTPD1 expression in PMA-differentiated THP-1 macrophages treated with vehicle, TNF-α, U0126, or TNF-α plus U0126.

B, Representative western blot images and quantification of p-ERK, t-ERK, MERTK, GAS6, IGF1, and ENTPD1 protein levels in PMA-differentiated THP-1 macrophages treated with vehicle, TNF-α, U0126, or TNF-α plus U0126.

C, Flow cytometric analysis of Annexin V and PI staining in SMCs after apoptosis induction. Annexin V⁺PI⁻ cells were quantified as apoptotic SMCs before co-culture with macrophages.

Data are presented as mean ± SEM (A-C). Statistical significance was determined using two-tailed unpaired t test (C), or one-way ANOVA with Tukey’s post hoc test (A-B). **P* ≤ 0.05; ***P* ≤ 0.01; ****P* ≤ 0.001; *****P* ≤ 0.0001; ns, not significant.


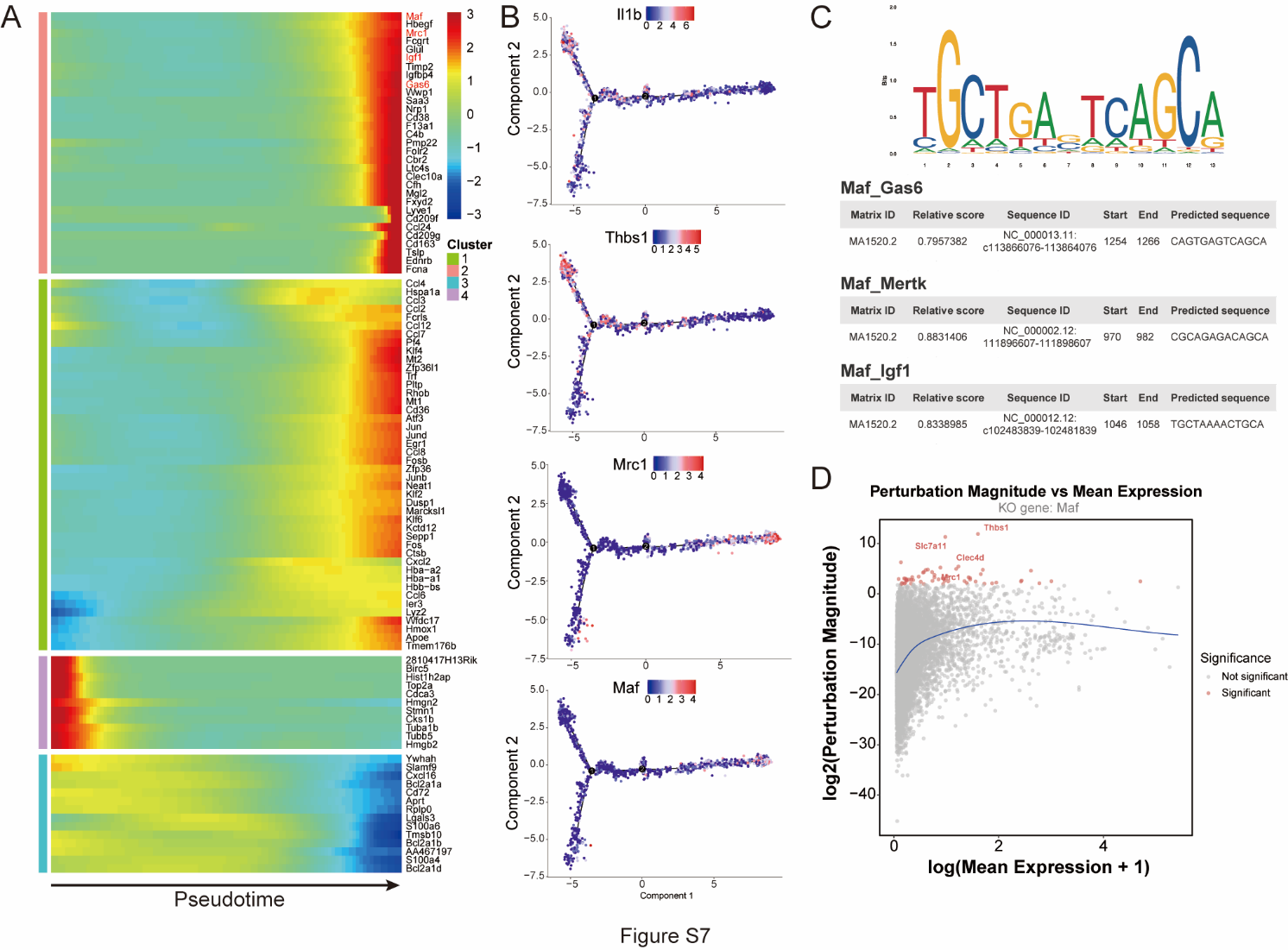


**Supplementary Figure 7. Analyses of macrophage state trajectories and Maf-associated perturbation.**

A, Heatmap showing the top 100 genes associated with macrophage pseudotime. Cells are ordered along the inferred pseudotime axis, and each row represents one gene.

B, Trajectory plots showing the expression patterns of representative genes, including Il1b, Thbs1, Mrc1, and Maf.

C, In silico prediction of Maf-binding sites within the 2kb upstream promoter regions of Gas6, Igf1, and Mertk using JASPAR. The highest-scoring predicted Maf-binding motif is shown for each gene.

D, MA plot showing gene perturbation after in silico Maf knockout in macrophages using the scTenifoldKnk package. Each dot represents one gene. The x-axis indicates log (mean expression + 1), and the y-axis indicates log2 perturbation magnitude. Significantly perturbed genes are highlighted.


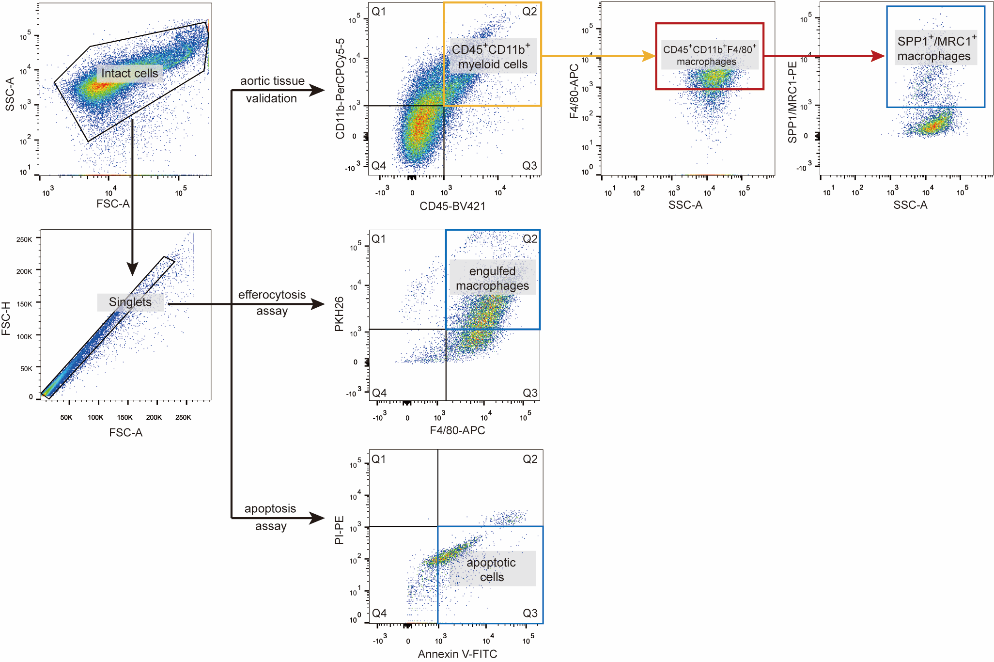


**Supplementary Figure 8. Flow cytometry gating strategies for macrophage validation, SMC apoptosis validation, and efferocytosis-associated uptake assay.**

Representative flow cytometry gating strategies used in this study. The major intact cell population was first selected using FSC-A and SSC-A to exclude debris and very small particles, followed by singlet gating using FSC-A and FSC-H to reduce doublets and aggregates. For aortic tissue validation, CD45^+^CD11b^+^ myeloid cells were selected from the singlet population, and macrophages were further defined as CD45^+^CD11b^+^F4/80^+^ cells. SPP1^+^ or MRC1^+^ macrophages were quantified within the CD45^+^CD11b^+^F4/80^+^ macrophage gate. For apoptosis validation before the efferocytosis assay, Annexin V and PI staining was used to quantify apoptotic SMCs after apoptosis induction. For the flow cytometry-based efferocytosis-associated uptake assay, F4/80^+^ macrophages were selected from the singlet population, and PKH26^+^ events within the F4/80^+^ macrophage gate were quantified as macrophages associated with PKH26-labeled apoptotic SMCs. FMO controls were performed for SPP1 and MRC1 to define target-marker positivity gates.

## Supplementary Tables

**Table S1.** PCR Primers for Quantitative RT-PCR.

| **Genes** | **Species** | **Forward Primer** | **Reverse Primer** |
| --- | --- | --- | --- |
| *Mertk* | M | CAGGGCCTTTACCAGGGAGA | TGTGTGCTGGATGTGATCTTC |
| *Igf1* | M | CTGGACCAGAGACCCTTTGC | GGACGGGGACTTCTGAGTCTT |
| *Gas6* | M | TGCTGGCTTCCGAGTCTTC | CGGGGTCGTTCTCGAACAC |
| *Entpd1* | M | AAGGTGAAGAGATTTTGCTCCAA | TTTGTTCTGGGTCAGTCCCAC |
| *Gapdh* | M | AGGTCGGTGTGAACGGATTTG | TGTAGACCATGTAGTTGAGGTCA |

**Major Resources Information**

**Table S2.** Experimental animals.

| **Species/Strain** | **Vendor or Source** | **Background Strain** | **Sex** |
| --- | --- | --- | --- |
| Wild type C57BL/6J | SPF (Beijing) Biotechnology Co., Ltd., China | C57BL/6J | Male |

**Table S3.** Cultured Cells and related reagents.

| **Cells or reagents** | **Vendor or Source** | **Catalog #** |
| --- | --- | --- |
| RAW 264.7 | Procell | CL-0190 |
| Human Aortic Smooth Muscle Cells | Procell | CP-H081 |
| THP-1 | Procell | CL-0233 |
| FBS | Gibco | 10099141C |
| P/S | Sigma | V900929 |
| PMA | MCE | HY-18739 |
| RPMI-1640 medium | Gibco | C11875500BT |
| DMEM, high glucose | Gibco | 11965092 |

**Table S4.** Antibodies.

| **Target antigen** | **Vendor or Source** | **Clone** | **Catalog #** |
| --- | --- | --- | --- |
| p44/42 MAPK (Erk1/2) (WB) | Cell Signaling Technology | Polyclonal | 9102S |
| Phospho-p44/42 MAPK (Erk1/2) (WB) | Cell Signaling Technology | Monoclonal | 4370S |
| ENTPD1 (WB) | Proteintech | Polyclonal | 14211-1-AP |
| IGF1 (WB) | Proteintech | Polyclonal | 28530-1-AP |
| GAS6 (WB/IF) | Proteintech | Polyclonal | 13795-1-AP |
| MERTK (WB/IF) | Proteintech | Polyclonal | 27900-1-AP |
| CDCA8 (IF) | Proteintech | Polyclonal | 12465-1-AP |
| MRC1 (IF/FC) | Proteintech | Monoclonal | 83485-1-RR |
| SPP1 (IF/FC) | Proteintech | Polyclonal | 22952-1-AP |
| THBS1 (IF) | Proteintech | Polyclonal | 18304-1-AP |
| CD68 (IF) | Invitrogen | Monoclonal | 14-0688-82 |
| CD36 (IF) | Proteintech | Monoclonal | 66395-1-Ig |
| CD45 CoraLite® Plus 405 (FC) | Proteintech | Monoclonal | CL405-98035 |
| CD11b PerCP-Cyanine5.5 (FC) | Proteintech | Monoclonal | CPY5-65055 |
| F4/80 CoraLite® Plus 647 (FC) | Proteintech | Monoclonal | CL647-98236 |
| Goat anti-rabbit IgG (HRP-conjugated, WB) | Proteintech | NA | SA00001-2 |
| Goat anti-mouse IgG  (HRP-conjugated, WB) | Proteintech | NA | SA00001-1 |
| CoraLite594 - conjugated Goat Anti-Rabbit IgG (IF) | Proteintech | NA | SA00013-4 |
| CoraLite488-conjugated Goat Anti-Mouse IgG (IF) | Proteintech | NA | SA00013-1 |

WB: western blot; IF: immunofluorescence; FC: flow cytometry.

**Table S5.** Other important reagents.

| **Name** | **Vendor or Source** | **Catalog #** |
| --- | --- | --- |
| FlexAble 2.0 CoraLite® Plus 555 Antibody Labeling Kit for Rabbit IgG | Proteintech | KFA502 |
| TSA Fluorescence Kit (7-color) | Panovue | 10268100050 |
| TRIzol | Invitrogen | 15596018 |
| PrimeScript RT Reagent Kit with gDNA Eraser | TaKaRa | RR047A |
| TB Green Premix Ex Taq II (Tli RNaseH Plus) | TaKaRa | RR820A |
| BCA Protein Assay Kit | Beyotime | P0012 |
| PVDF membrane (0.45 μm) | Thermo Scientific | 88518 |
| One Step TUNEL Apoptosis Assay Kit | Beyotime | C1089 |
| PKH26 Red Cell Membrane Staining Kit | Solarbio | D0030 |
| Apoptosis Inducer Kit (TNF-α + SM-164) | Beyotime | C0006S |
| UNC2025 | MCE | HY-12344 |
| U0126 | MCE | HY-12031A |
| Cytochalasin D | MCE | HY-N6682 |
| TNFα | Peprotech | 300-01A-10UG |
| Collagenase type I | Gibco | 17100-017 |
| Collagenase type XI | Sigma | C7657 |
| Hyaluronidase type I-S | Sigma | H3506 |
| DNase I | Sigma | DN25 |
| Elastase type I | Sigma | E1250 |
